# Mechanisms of SARS-CoV-2 Inactivation Using UVC Laser Radiation

**DOI:** 10.1021/acsphotonics.3c00828

**Published:** 2023-12-26

**Authors:** George Devitt, Peter B. Johnson, Niall Hanrahan, Simon I. R. Lane, Magdalena C. Vidale, Bhavwanti Sheth, Joel D. Allen, Maria V. Humbert, Cosma M. Spalluto, Rodolphe C. Hervé, Karl Staples, Jonathan J. West, Robert Forster, Nullin Divecha, Christopher J. McCormick, Max Crispin, Nils Hempler, Graeme P. A. Malcolm, Sumeet Mahajan

**Affiliations:** † School of Chemistry, Faculty of Engineering and Physical Sciences, University of Southampton, Highfield, Southampton SO17 1BJ, United Kingdom; ‡ School of Biological Sciences, Faculty of Environmental and Life Sciences, University of Southampton, Highfield, Southampton SO17 1BJ, United Kingdom; § Institute for Life Sciences, University of Southampton, Highfield, Southampton SO17 1BJ, United Kingdom; ∥ Clinical and Experimental Sciences, Faculty of Medicine, University of Southampton, Sir Henry Wellcome Laboratories, University Hospital Southampton, Southampton SO16 6YD, United Kingdom; ⊥ University of Cambridge, MRC Toxicology Unit, Cambridge, CB2 1QR, United Kingdom; # Wessex Investigational Sciences Hub, University of Southampton Faculty of Medicine, Southampton General Hospital, Southampton SO16 6YD, United Kingdom; ∇ Southampton NIHR Biomedical Research Centre, Southampton General Hospital, Southampton SO16 6YD, United Kingdom; ○ Cancer Sciences, Faculty of Medicine, University of Southampton, Southampton SO16 6YD, United Kingdom; ¶ M Squared Lasers, Limited, 1 K Campus, West of Scotland Science Park, Glasgow, G20 0SP, United Kingdom; ▲ Department of Biotechnology, Inland Norway University of Applied Sciences, Holsetgata 22, N-2317 Hamar, Norway

**Keywords:** COVID-19, ribonucleic acid, protein conformation, Raman spectroscopy, disinfection

## Abstract

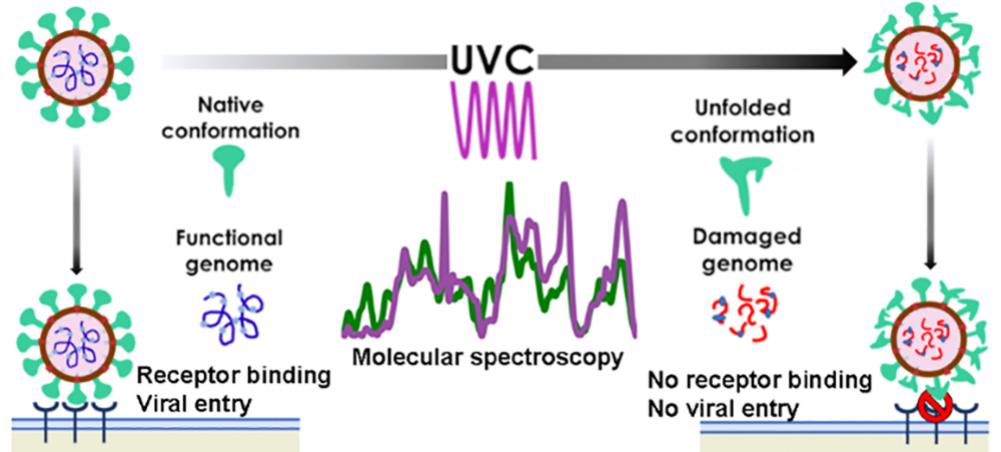

Severe acute respiratory syndrome coronavirus 2 (SARS-Cov-2) has had a tremendous impact on humanity. Prevention of transmission by disinfection of surfaces and aerosols through a chemical-free method is highly desirable. Ultraviolet C (UVC) light is uniquely positioned to achieve inactivation of pathogens. We report the inactivation of SARS-CoV-2 virus by UVC radiation and explore its mechanisms. A dose of 50 mJ/cm^2^ using a UVC laser at 266 nm achieved an inactivation efficiency of 99.89%, while infectious virions were undetectable at 75 mJ/cm^2^ indicating >99.99% inactivation. Infection by SARS-CoV-2 involves viral entry mediated by the spike glycoprotein (S), and viral reproduction, reliant on translation of its genome. We demonstrate that UVC radiation damages ribonucleic acid (RNA) and provide in-depth characterization of UVC-induced damage of the S protein. We find that UVC severely impacts SARS-CoV- 2 spike protein’s ability to bind human angiotensin-converting enzyme 2 (hACE2) and this correlates with loss of native protein conformation and aromatic amino acid integrity. This report has important implications for the design and development of rapid and effective disinfection systems against the SARS-CoV-2 virus and other pathogens.

## Introduction

The COVID-19 pandemic caused by the severe acute respiratory syndrome coronavirus-2 (SARS-CoV-2)^1,2^ spreads via nosocomial, public, and work-place based infections.^3^ Transmission is thought to be direct via respiratory droplets or indirect via fomites and has led to increased interest in viral disinfection, including the use of ultraviolet (UV) light to inactivate virus in aerosols and on surfaces. The majority of studies into the effects of UV have focused on the effects of UVA (400–320 nm) and UVB (320–280 nm) due to the prevalence of these wavelengths in sunlight^4^ and the availability of light sources in these ranges. More recently UVC (280–200 nm) has seen increased interest as a method for viral inactivation in blood plasma extracts.^5,6^ Different UVC spectral regions present different properties with regards to their depth of penetration which can both be advantageous, inability to penetrate deeply into biological tissues can potentially render them safer, or disadvantageous since they will only disinfect surfaces.^7^ Short wavelength UVC (<240 nm) shows increased efficacy for inactivation of MS2 phage,^8^ influenza,^9^ human coronaviruses,^10^ and damaging adenoviral proteins.^11^ These effects have been attributed to the absorption peak of proteins around 230 nm^12,13^ but the effect of UVC on viral protein structure and function is not yet understood. This is important given the critical role that proteins play in viral cell entry.^14^

Coronaviruses depend on a number of structural proteins for virus particle formation that include spike (S), membrane (M), envelope (E), and nucleocapsid (N) proteins. S forms trimeric projections from the surface of the virion, giving it its characteristic crown-like appearance. Each S subunit contains an S1 and S2 domain, mediating cell attachment and membrane fusion, respectively. SARS-CoV-2 S has a receptor binding domain (RBD) within the C-terminal region of S1 that is responsible for binding human angiotensin-converting enzyme 2 (hACE2); an interaction driving SARS-CoV-2 cell tropism.^15^ Cleavage of the S, both at the S1–S2 boundary by furin, and within the S2 subunit by TMPRSS2 and cathepsin-L, primes the protein for membrane fusion.^16−19^ Owing to its dual role in mediating receptor binding and membrane fusion, we hypothesized S protein damage may be an effective route to viral inactivation.

Native conformation and structural integrity of proteins are required for correct function. The S protein-hACE2 binding involves a key sequence of amino-acid residues^20−23^ constituting the receptor binding domain (RBD). The RBD conformation contains a twisted five-stranded antiparallel β sheet with short connecting helices.^24,25^ Further, within this core, 2 α-helices and 2 β-strands, containing multiple key aromatic residues, and a disulfide bridge, form the receptor binding motif (RBM).^22^ Aromatic residues (tryptophan, tyrosine, and phenylalanine) absorb UVC light at 280, 275, and 258 nm, respectively, and disulfide bonds absorb at 260 nm.^26^ Therefore, the RBM and the spike protein as a whole, along with other viral proteins, is a potential target for UVC-induced damage.

In this work we demonstrate UVC inactivation of SARS-CoV-2 virus and its dose dependence. To understand the mechanism of UVC inactivation we use two UVC wavelengths (266 nm, near UVC, 227 nm, far UVC) from solid-state continuous wave lasers. 266 nm radiation is strongly absorbed by nucleic acids^27^ and by the aromatic rings of amino acid side chains while 227 nm is absorbed less than 266 nm by nucleic acids, but more strongly by proteins.^28^ We explore the dose dependent damage to viral RNA and its scaling with genome size. We probe in-depth the effect of UVC on the SARS-CoV-2 S protein using binding assays, morphological characterization and molecular spectroscopy. UVC irradiation of SARS-CoV-2 S protein severely affected its ability to bind to hACE2 which correlated well to the observed changes in protein conformation and oxidation of aromatic residues. We postulate that the UVC induced damage to proteins, in combination with that to RNA, is an important contributor toward the inactivation of the SARS-CoV-2 virus with immediate implications for wavelength and dose selection for preventing transmission of Covid-19 and other airborne pathogens.

## Results and Discussion

### SARS-CoV-2 Inactivation Using 266 nm Laser

**Figure 1:**
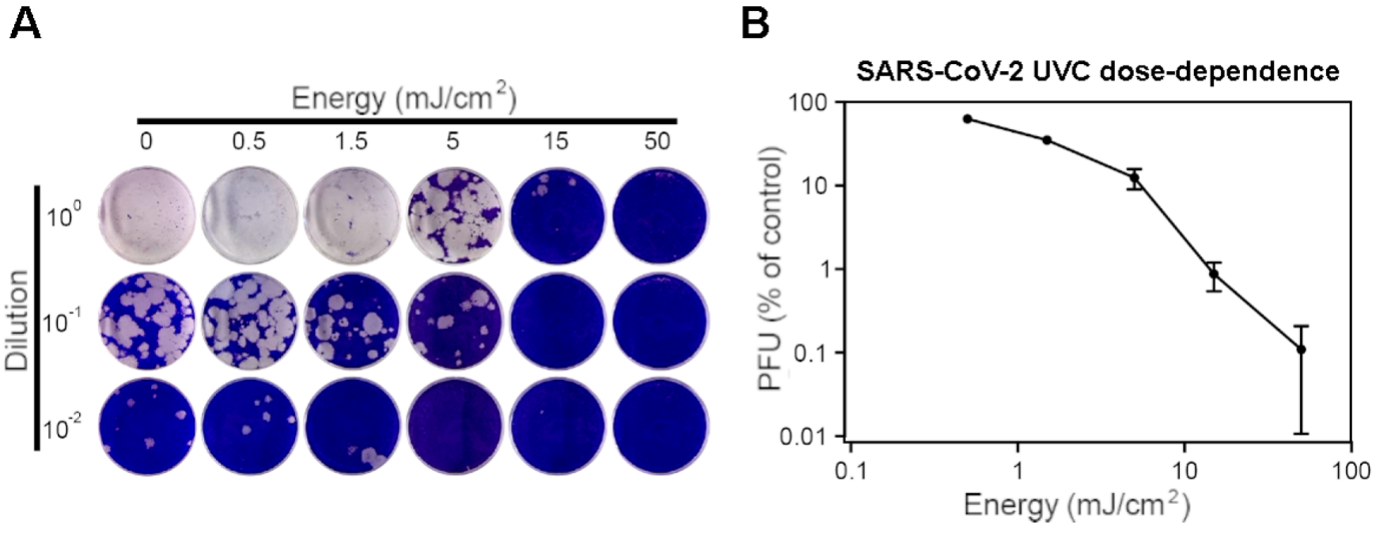
SARS-CoV-2 virus surface inactivation by 266 nm UVC continuous wave laser. (A) Representative set of images from a plaque assay showing the dose (horizontal) and serial dilution (vertical). (B) Dose-dependence of SARS-CoV-2 virus to 266 nm UVC radiation, from two independent experiments performed in triplicate.

To examine the impact of UVC on SARS-CoV-2 infectivity, virus was dried on to polystyrene surfaces which were then exposed to 266 nm irradiation at 0.5 mW/cm^2^ for between 1 and 100 s. The exposed virus samples were resuspended and assessed by plaque assay. A clear dose dependent response was seen, with the highest dose (50 mJ/cm^2^) leading to 99.89% inactivation (Figure 1A,B and Supporting Information, SI, Figure S1). In a separate experiment (operational constraints in the containment level 3 laboratory meant that altering radiant power within one experiment was not feasible) following a 25 mJ/cm^2^ (1s, 25 mW/cm^2^) dose, only a single infectious virion across 3 technical repeats was recovered, equating to 99.97% inactivation. Doses of 75 mJ/cm^2^ (25 mW, 3s) or greater reduced infectivity below the limit of detection of 0.01% (SI Figure S2). Thus, surface decontamination of SARS-CoV-2 by 266 nm UVC can be achieved within seconds at modest UVC doses.

**Figure 2:**
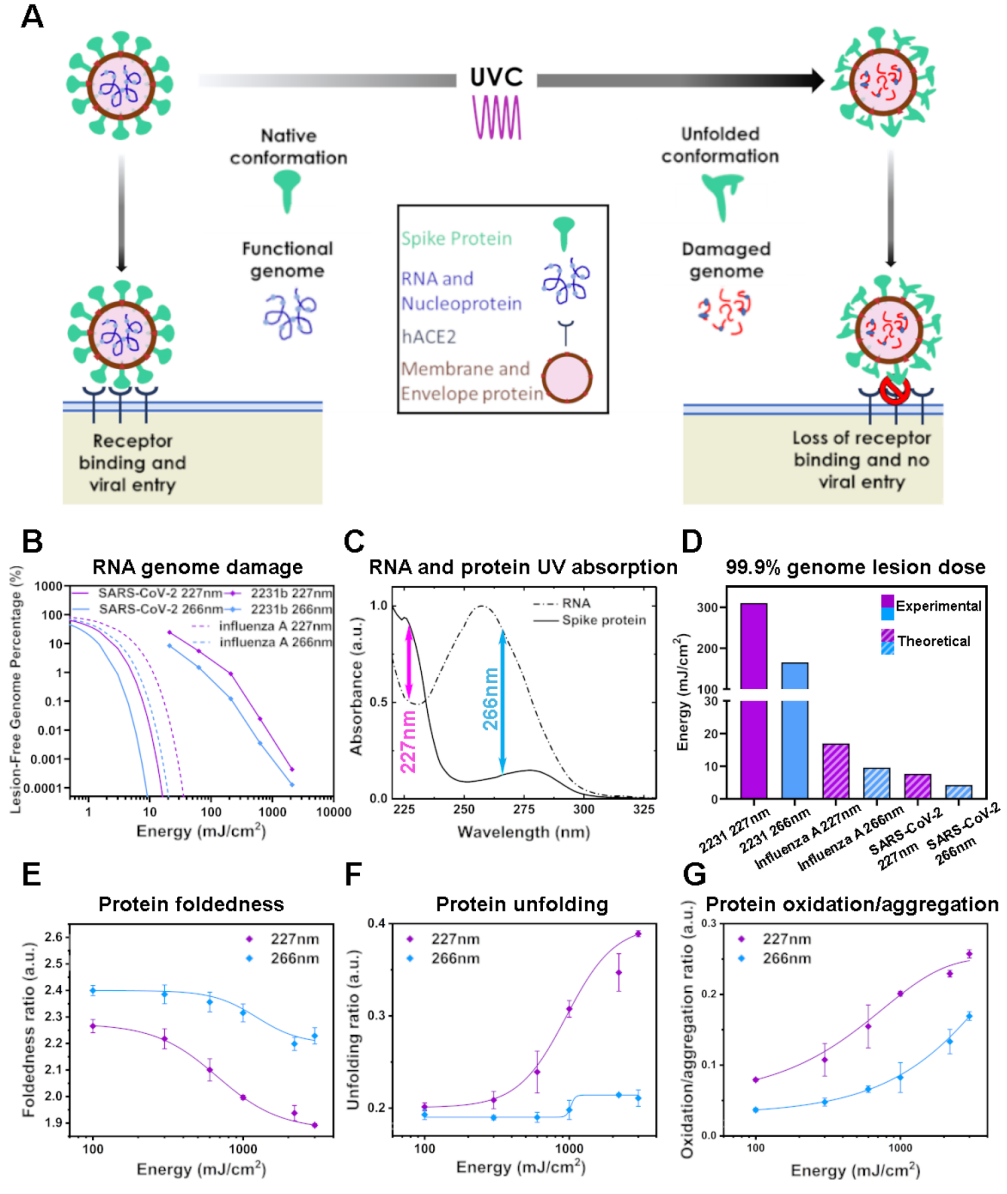
UVC dose dependence of ssRNA and rSARS-CoV-2 S protein integrity. (A) Schematic depicting the effect of UVC radiation on SARS-CoV-2 cellular entry and reproduction. (B) Dose- dependent RNA inactivation. Figure depicts RT-qPCR product off ∼2kb RNA template, and projected 30kb RNA template as a percentage of nonirradiated RNA control. *n* = 3, plotted data represents mean and SD from 6 cycle threshold readings. (C) UV absorption spectrum of rSARS-CoV-2 S. Arrows indicate UVC laser wavelengths used in this study. (D) Experimentally determined 227 and 266 nm UV dose requirements for 99.9% replication inhibition from the 2231 base MS2 amplicon, and theoretical model extrapolated using Poisson statistics for larger 13 588 and ∼30 kb SARS-CoV-2 genomes. (E) Dose-dependence of rSARS-CoV-2 S foldedness ratio to UVC radiation determined by UV–vis absorption spectroscopy. Foldedness ratio = A280/A275 nm + A280/A258 nm. (F) Dose-dependence of rSARS-CoV-2 S unfolding ratio to UVC radiation determined by UV–vis absorption spectroscopy. Unfolding ratio = A280/A230 nm. (G) Dose-dependence of SARS-CoV-2 S oxidation/aggregation ratio to UVC radiation determined by UV–vis absorption spectroscopy. Oxidation/aggregation ratio = A320/A280 nm. *n* = 2, the plotted data represent the average and SD from 10 absorbance readings for native rSARS-CoV-2 S and 3–4 absorbance readings per UVC condition.

For comparison the UVC inactivation dose observed is higher than the ≤2 mJ/cm^2^ reported for human coronaviruses alpha HCoV-229E, beta HCoV-OC43^9^ and H1N1 influenza.^10^ This could be due to the use of 222 nm radiation in those studies, or due to a much shorter path length (≤1 μm aerosols vs dried 25 μL media droplets used here). Additionally, the above coronaviruses use human aminopeptidase N^29^ and sialic acid sugars^30^ as a receptor for cell entry, while SARS-CoV-2 virus spike protein is optimized for binding hACE2.^31^ Viral cell entry, mediated by the spike protein, and replication, facilitated by the genome, are both essential parts of the infection processes (Figure 2A). Therefore, we explored the effect of UVC on macromolecular components involved in SARS-CoV-2 viral infection.

### UVC Damage of RNA

227 and 266 nm UVC wavelengths were used to test the effect on RNA integrity using the MS2 ssRNA viral genome (3.57 kb) and an RT-qPCR assay.^8^ Here we define RNA damage as that sufficient to prevent in vitro reverse transcriptase progression, however, we note that host ribosomal sensitivity to damaged RNA may differ. Damage therefore results in less cDNA template for the subsequent PCR reaction. Our assay could detect RNA at concentrations up to 6 orders of magnitude below the stock concentration. We found that both wavelengths damaged RNA, in a dose dependent manner (Figure 2B, 99.9% reduction dose: 227 nm, 285 mJ/cm^2^; 266 nm, 180 mJ/cm^2^), but 266 nm was more effective, presumably as a result of the strong absorption by RNA around 260 nm (Figure 2C).

In our assay the PCR primers amplified targets 925 bases and 2231 bases distal from the primer used for first strand synthesis. Thus, our assay probed RNA integrity only for these lengths, while the SARS-CoV-2 genome is ∼30kb in length. Using a probabilistic interpretation involving the Poisson statistic that considers that UV-mediated RNA lesion events are proportional to genome length we modeled our results to predict the required dose for a three log reduction in the SARS-CoV-2 genome, which we assume will result in virus inactivation (Figure 2B,D, SI Figure S3). To damage 99.9% of SARS-CoV-2 genome the model predicts a dose of only 7.7 mJ/cm^2^ at 227 nm or 4.25 mJ/cm^2^ at 266 nm. These are not dissimilar to the dose needed to inactivate other human coronaviruses using similar wavelengths.^10^ However, the viral inactivation dose observed for SARS-CoV-2 was ∼11.7 times higher (∼50 mJ/cm^2^ at 266 nm, Figure 1B). Proteins also absorb UVC radiation in this spectral range (Figure 2C). Hence, we next investigated the possibility that UVC induced damage to proteins in the virus particles, with implications for structural integrity and capacity for cell entry.

### UVC Damage of SARS-CoV-2 Spike Protein

We expressed solubilized and stabilized recombinant SARS-CoV-2 spike protein (rSARS-CoV-2 S). The rSARS-CoV-2 S variant replaces the furin cleavage site with a “GSAS” linker and, along with several other mutations, helps to stabilize the trimeric structure of the spike protein.^25^ The mutations are required to maintain native-like structure and we note that this increased stabilization likely sets a higher threshold for UVC-mediated inactivation. rSARS-CoV-2 S has two peaks in its UV–vis absorption spectrum, which can be targeted by the two wavelengths used in this study (Figure 2C). We established a UVC dose–response for each wavelength for rSARS-CoV-2 S protein integrity using UV–visible absorption spectra as a readout (Figure 2E–G). The conformational state of a protein affects its absorption properties, thus protein unfolding, oxidation and aggregation are all reflected by changes in the absorption spectrum (31). We observed a dose-dependent decrease in the foldedness ratio of rSARS-CoV-2 S (A280 nm/A275 nm + A280 nm/A258 nm)^26^ with increasing dose of UVC radiation, with 227 nm producing a greater dose matched effect than 266 nm (Figure 2E). The A230 nm peak is sensitive to protein secondary structure, with an increase in intensity observed upon protein unfolding.^32^ We observed a large dose-dependent increase in the A230 nm/A280 nm ratio for rSARS-CoV-2 S in response to 227 nm, with a subtle increase in response to 266 nm irradiation (Figure 2F). This suggests that 227 nm irradiation damages secondary structure more than 266 nm irradiation. Similarly, the A320 nm/A280 nm ratio increases upon protein aggregation^33^ or upon oxidation of tryptophan to *n*-formylkynurenine (NFK).^34^ We detected a UVC dose-dependent increase in A320 nm/A280 nm ratio (Figure 2G), again consistent with loss of native protein conformation. Taken together S protein unfolding and conformational loss due to UVC exposure are predicted to impair functionality.

### UVC Induced Loss of rSARS-CoV-2-hACE2 Binding

In order to correlate the changes caused by UVC treatment to the functionality of the rSARS-CoV-2 spike protein we selected low (100 mJ), medium (600 mJ), and high (2200 mJ) UVC doses (SI Figure S4) and performed surface plasmon resonance (SPR) binding assays. To determine the ability of rSARS-CoV-2 S to bind hACE2, both with and without UVC treatment, the protein was expressed with a C-terminal HisTag which allows for both purification and also binding to a nickel-coated SPR sensor surface.

**Figure 3:**
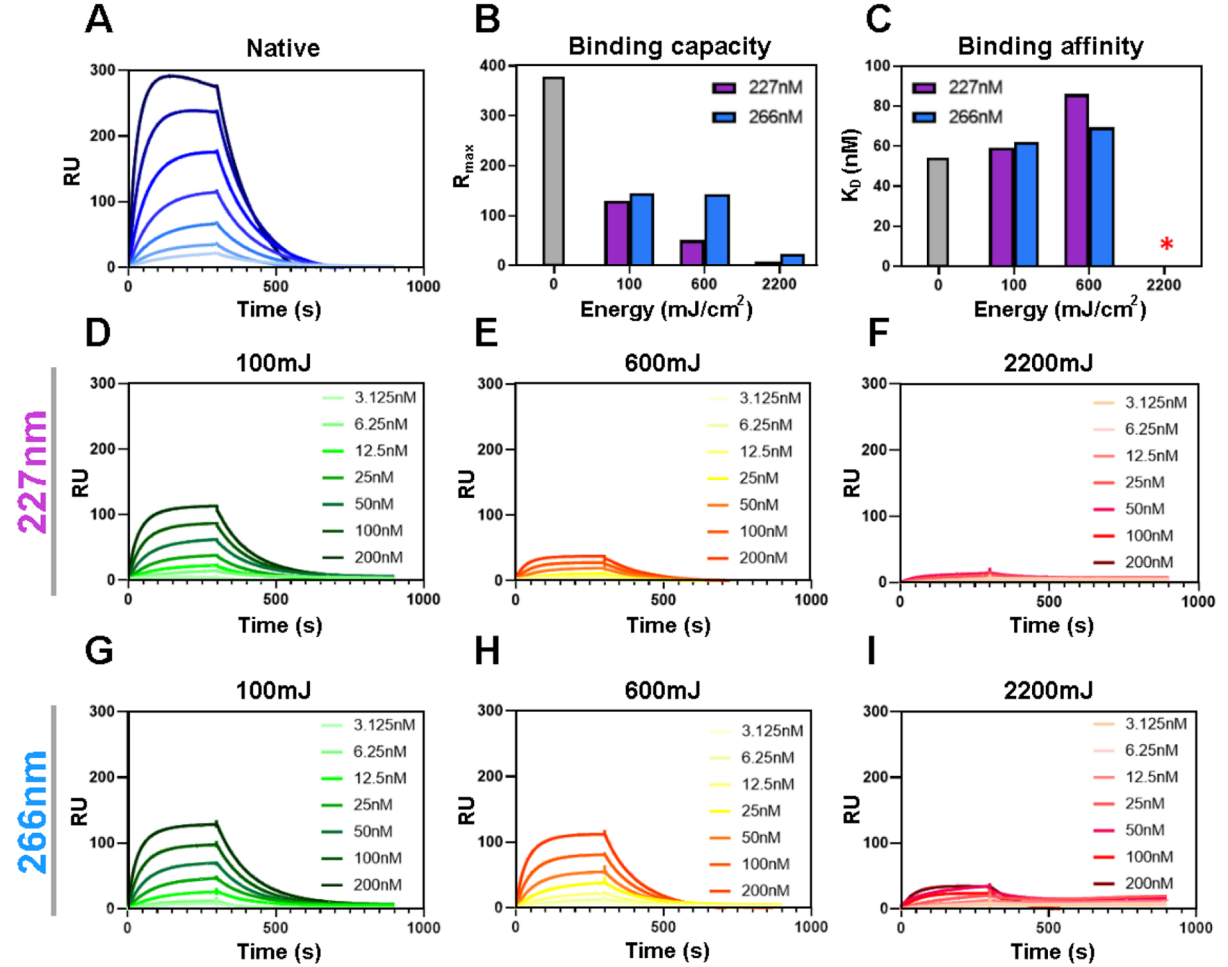
Surface plasmon resonance (SPR) analysis of rSARS-CoV-2 trimeric spike glycoprotein shows the impacts of UVC treatment on hACE2 binding. (A) Sensogram depicting the binding of solubilized recombinant SARS-CoV-2 to serial dilutions of the hACE2 receptor with dilutions ranging from 200 nM to 3.125 nM. Data represent an average of three analytical repeats. RU = resonance units (proportional to the number of SARS-CoV-2 S protein molecules bound to the surface). (B) Comparison of the calculated Rmax (the maximum observed binding signal) values for each UVC treatment condition expressed as percentage of the nonirradiated protein. (C) Binding affinity, measured as equilibrium dissociation constant or *K*_D_ values, for all of the SPR experiments performed. This parameter was calculated using a 1:1 binding model using Biacore Evaluation software. The response for the 2200 mJ dose for 227 and 266 nm was not sufficient to accurately determine KD. (D–I) SPR experiments were performed identically to panel A except an equal amount of S protein was treated with the dosage indicated.

To measure binding affinity, serial dilutions of hACE2 were sequentially flowed over the rSARS-CoV-2 S protein-coated chip and the response measured (Figure 3A and 3B). Untreated S protein bound hACE2 with an equilibrium dissociation constant (KD) of 54 nM (Figure 3C and SI Table S1). This value represents the binding affinity, with smaller values reflecting higher binding affinities between rSARS-CoV-2 S protein and hACE2. Using a flow rate of 10 μL/min for 240 s resulted in a calculated maximum observed binding signal (*R*_max_) of 377.4. This value reflects the amount of rSARS-CoV-2 S protein which has successfully bound to the chip. Both UVC wavelengths, 227 and 266 nm, resulted in an increase in KD and a concurrent decrease in *R*_max_ (Figure 3D–I) indicating higher dissociation and reduced binding. At the highest dose tested (2200 mJ/cm^2^), the *R*_max_ was only 2% compared to that of untreated rSARS-CoV-2 S protein for 227 nm and 6% for 266 nm irradiation which are similar to the negative control. This is likely due to degradation of the S protein, inhibiting hACE2 binding. The 600 mJ dose resulted in both an increase in *K*_D_, reflecting a decrease in affinity of rSARS-CoV-2 S protein for hACE2, and an *R*_max_ decrease, with the 227 nm wavelength being more effective than the 266 nm wavelength. The 100 mJ dose resulted in a smaller increase in *K*_D_ and decrease in *R*_max_ compared to the 600 mJ dose, but at this dosage both 227 and 266 nm wavelengths performed similarly. While UVC dose dependent reduction in binding is clear, the largest percentage decrease in binding capacity occurs within 100 mJ itself (Figure 3B). The higher dose required for a three log reduction of the recombinant SARS-CoV-2 spike protein in comparison to the virus may be due to the synergistic effect of UVC induced RNA damage, as well as differences in stability of the recombinant and wild-type viral spike protein. Technical differences in each experiment may also contribute to this discrepancy; the SARS-CoV-2 virus was dried in culture media onto the wells of polystyrene plates (and exposed from the top without the lid in place), while the recombinant protein was irradiated in PBS solution through quartz slides. This may have resulted in differences in the absorption and/or reflection of the UVC light for each sample. That UVC damages the spike protein, reduces its binding ability and thus will diminish viral entry is clear. Therefore, UVC wavelengths, which damage both viral entry and replication, are likely to be very powerful disinfectants for viruses such as SARS-CoV-2.

### UVC Radiation Has Distinct Effects on rSARS-CoV-2 S Conformation

In order to understand the reduction of rSARS-CoV-2 S function, we investigated the global changes in protein conformation induced by UVC. We first describe the effect with 227 nm and then 266 nm.

**Figure 4:**
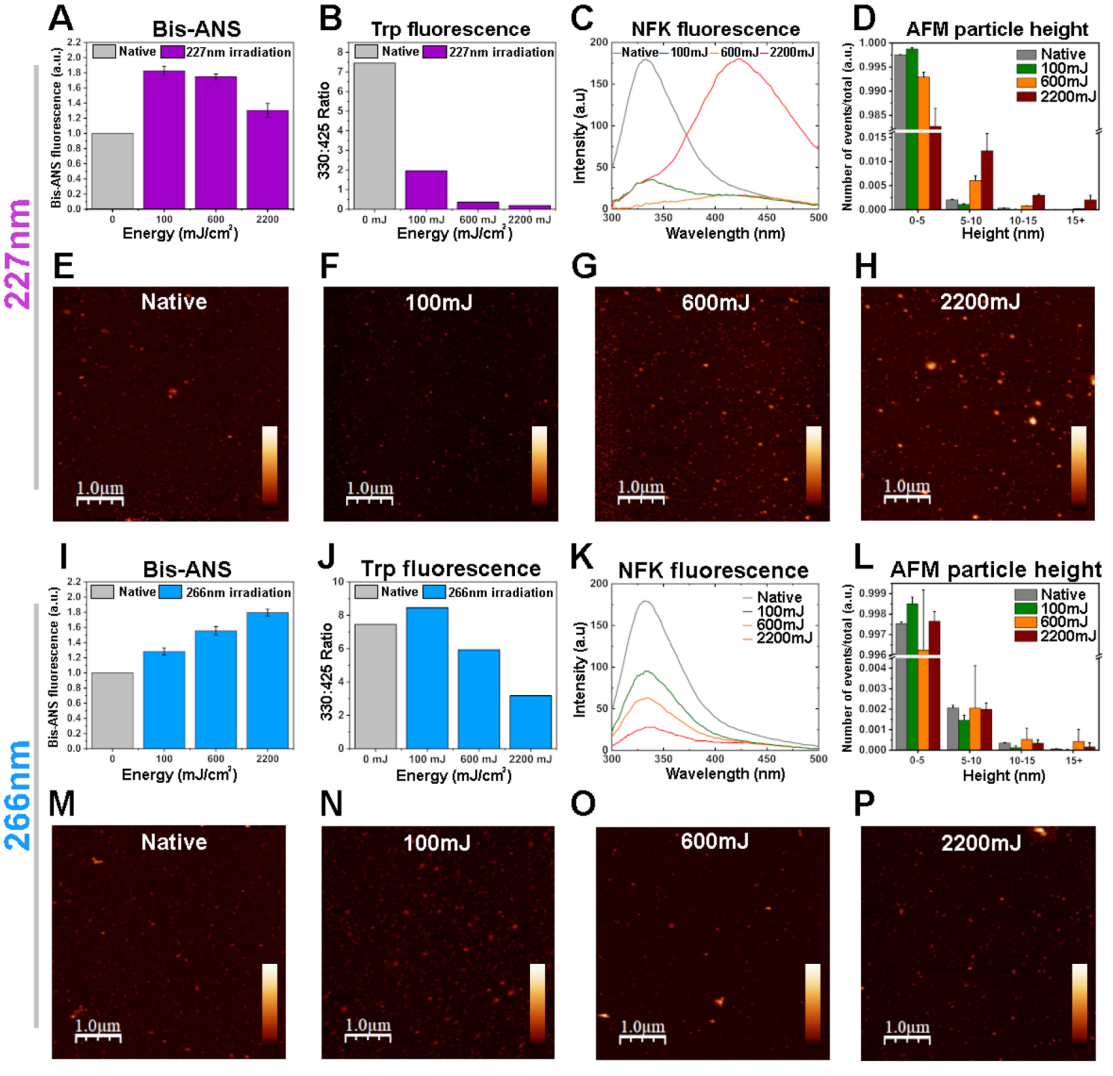
UVC dose-dependent loss of rSARS-CoV-2 spike protein conformation. Readouts of rSARS-CoV-2 spike protein conformation induced by 227 nm (A–H) or 266 nm (I–P) radiation (A,I) Bis-ANS binding measured by fluorescence emission at 490 nm. *n* = 2, the plotted data represent the average and SD from 4 fluorescence readings. (B,J) Fluorescence emission ratio 330/425 nm representing tryptophan/NFK fluorescence. (C,K) Fluorescence emission spectra from 300 to 500 nm representing changes in intrinsic tryptophan fluorescence. *n* = 1, plotted data represents the average from 3 fluorescence readings. (D,L) Particle height analysis of atomic force microscopy (AFM) images. 3–4 images were used for each condition. *n* = 2, the plotted data represent the average and SD from 2 to 4 AFM images. (E–H, M–P) Representative tapping-mode AFM images of SARS-CoV-2 spike protein bound to a mica surface after irradiation with 0 mJ (E,M), 100 mJ (F,N), 600 mJ (G,O), 2200 mJ (H,P). Images are 5 × 5 μm^2^, scale bars represent 1 μm and *z*-scale is equal to 0–30 nm.

Hydrophobic residues buried within the native protein structure can be exposed upon protein unfolding. Hence, to measure surface hydrophobicity we used a Bis-ANS (4,4′-dianilino-1,1′-binaphthyl-5,5′-disulfonic acid dipotassium salt) binding assay. Increased Bis-ANS binding and fluorescence was observed with 227 nm UVC irradiation (Figure 4A), however, this increase in hydrophobicity became smaller with increasing doses. This may be because Bis-ANS has lower affinity for oxidized proteins.^35^ Therefore, we probed rSARS-CoV-2 S for oxidation sensitive tryptophan residues (reduced tryptophan fluoresces at 330 nm and its oxidized product, *N*-formyl kynurenine (NFK), fluoresces at 425 nm). We observed a dose-dependent decrease in the 330 nm/425 nm emission ratio (Figure 4B). This decreasing ratio is primarily due to a loss in tryptophan fluorescence, with a large increase in NFK fluorescence only occurring at the highest dose (Figure 4C).

Protein unfolding and the exposure of hydrophobic residues can lead to protein aggregation.^36^ We therefore used atomic force microscopy (AFM) to probe the protein morphology. Particle height analysis was performed on AFM images to quantify the number of protein aggregates induced by 227 nm irradiation (Figure 4D). Medium and high doses of 227 nm irradiation induced protein aggregation, with a transition from smaller structures (<5 nm) to larger assemblies (5–50 nm). The morphology of these assemblies can be observed in the representative AFM images, which show an increase in amorphous aggregates after exposure to medium and high doses of 227 nm radiation (Figures 4E–H, SI Figure S5).

Interestingly, higher doses of 266 nm radiation did not induce the same effects as 227 nm radiation on protein conformation. We observed a dose-dependent increase in Bis-ANS binding (Figure 4I), in contrast to 227 nm radiation. The same trends were observed using bovine serum albumin (BSA) irradiated by 227 and 266 nm wavelengths (SI Figure S6). In order to assess whether this difference was due to the lower efficiency of the 266 nm radiation on damaging protein conformation, we irradiated rSARS-CoV-2 S with a dose of 10J (∼4.5 fold greater than the high dose), which resulted in a further increase in Bis-ANS binding (SI Figure S7). In line with this, the 330 nm/425 nm ratio from tryptophan fluorescence showed a lesser decrease after 266 nm irradiation in comparison to 227 nm irradiation (Figure 4J), with no visible NFK fluorescence being observed at 425 nm (Figure 4K). Further, 266 nm radiation does not cause the same amount of amorphous protein aggregation as 227 nm radiation at the doses used (Figure 4L–P). Together this shows that both wavelengths can cause protein damage and unfolding, however 227 nm irradiation of SARS-CoV-2 causes distinctly higher tryptophan oxidation and amorphous protein aggregation compared to 266 nm irradiation, which correlates with a greater reduction in hACE-2 binding.

### 227 nm UVC Damages rSARS-CoV-2 Secondary and Tertiary Structure

To determine the specific molecular mechanisms of UVC-induced unfolding of rSARS- CoV-2 S for 227 and 266 nm radiation, we probed for vibrational changes using Raman spectroscopy. The Raman spectrum includes several regions sensitive to protein conformation: Amide I at ∼1650 cm^–1^, an established marker of secondary structure; disulfide bonding at ∼500 cm^–1^; and aromatic ring vibrations throughout the Raman spectrum, sensitive to tertiary structures.^37^

**Figure 5:**
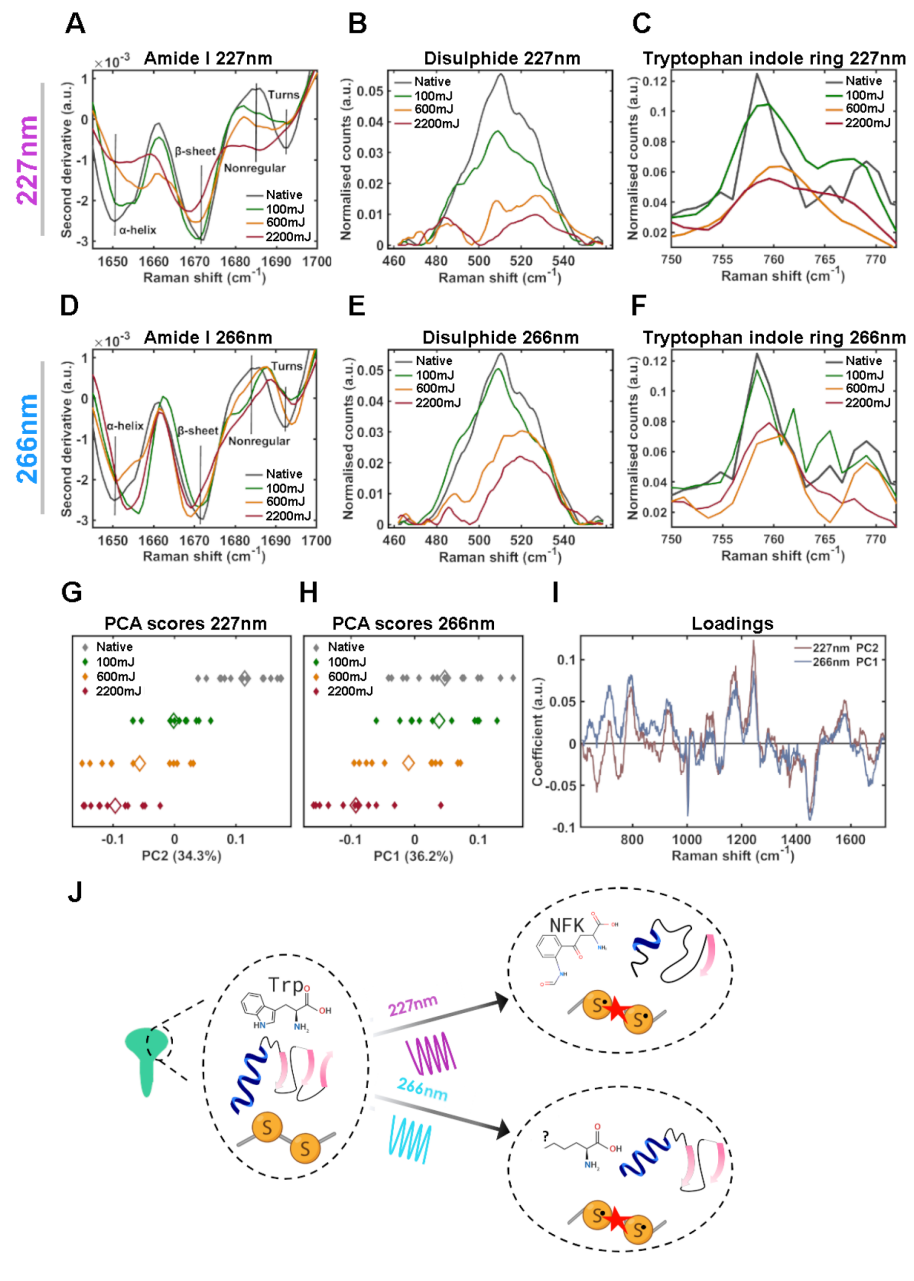
Mechanism of UVC-induced rSARS-CoV-2 S conformational damage. Raman spectra of rSARS-CoV-2 S protein following irradiation by 227 nm (A–C) or 266 nm (D–F) light. (A,D) Amide I second derivative spectra from 1645 to 1700 cm^–1^ (B,E) Disulfide region spectra from 462 to 558 cm^–1^. (C,F) Tryptophan indole ring spectra from 750 to 772 cm^–1^. (G–H) 1-Dimensional principle component analysis (PCA) scores plot of Raman spectra for 227 nm (G) and 266 nm (H) irradiated rSARS-CoV-2 S. Each solid diamond represents the PC score of a single spectrum. Hollow diamonds represent mean score. (I) PC loadings spectra representing the spectral variation responsible for the score across the given PC axis. (J) Schematic depicting conformational damage induced by 227 and 266 nm radiation determined by Raman spectroscopy. *n* = 2, plotted spectra represent the class means of 10–15 spectra per class.

We acquired Raman spectra of the untreated and UVC treated proteins and analyzed the above-mentioned regions of the spectrum (SI Figure S8). The second derivative of the Amide I spectra allows subtle changes to be highlighted and “hidden peaks” revealed.^38^ The second derivative spectrum for native rSARS-CoV-2 S (Figure 5A, gray trace) shows strong peaks at 1650 cm^–1^ (α-helix), 1672 cm^–1^ (β-sheet), and a small peak at 1693 cm^–1^ (turns). This is in line with the crystal structure of SARS- CoV-2 S.^25^ Upon irradiation with 227 nm wavelength, rSARS-CoV-2 S shows a dose dependent loss in α-helix and β-sheet vibrations, with a concurrent increase in intensity ∼1685 cm^–1^, corresponding to nonregular structure. 227 nm (100 mJ) causes loss of turn structures. 227 nm UVC irradiation also causes a dose-dependent decrease in the ∼509 cm^–1^ peak demonstrating a loss of disulfide bonds (Figure 5B). Thus, given that the SARS-CoV-2 RBM contains two β-strands, 2 α-helices and a disulfide bond,^22^ these data correlate well with the observed loss in hACE-2 binding (Figure 3).

There are a number of aromatic amino acid markers in the Raman spectrum, including those for phenylalanine, tyrosine and tryptophan. We observe a dose-dependent decrease in the tryptophan indole ring vibration 760 cm^–1^ peak with increasing 227 nm dose, as well as peak broadening, suggesting a loss in the ordered ring structure of tryptophan (Figure 5C) This suggests tryptophan oxidation^39^ and protein denaturation.^40^ Further evidence of protein denaturation is suggested by the dose-dependent increase in the ratio of 830 cm^–1^/850 cm^–1^ (SI Figure S9). This corresponds to the Fermi resonance vibration in tyrosine and indicates changes in the tyrosine hydrogen bonding environment^41^ that correlate well with the Bis-ANS data (Figure 4). Together, this suggests that irradiation of rSARS-CoV-2 S by 227 nm light causes a loss in secondary and tertiary structure, resulting in denaturation of the protein and is consistent with its loss of function.

### 266 nm UVC Primarily Damages SARS-CoV-2 Tertiary Structure

We similarly assessed conformational changes induced by 266 nm irradiation in the spike protein. While a dose-dependent loss in α-helix and β-sheet peak intensity was not observed (Figure 5D) an overall loss in the peak intensity of turns and an increase in nonregular structure was observed. Thus, unfolding occurs but to a lesser degree than with 227 nm irradiation, which is in agreement with UV–vis absorption results (Figure 2F). A dose-dependent decrease of the disulfide peak (Figure 5E, 509 cm^–1^), as well as in the tryptophan indole ring vibration peak (Figure 5F, ∼760 cm^–1^) were observed. Together, this suggests that irradiation of rSARS-CoV-2 S by 266 nm light causes a loss in native conformation, although secondary structural components are less effected when compared to 227 nm radiation. Disulfide bonds absorb weakly at ∼260 nm^42^ but excitation energy transfer (EET)^43^ from tryptophan and tyrosine can cause their cleavage.^44,45^ As both 227 and 266 nm radiation cause disulfide breakage in rSARS-CoV-2 S, it is likely that the cystine bonds are being targeted indirectly by EET due to their vicinity to Trp and Tyr residues.

### 227 and 266 nm UVC Degrade Aromatic Rings

S protein tyrosine residue (Y484) is centrally involved in hACE-2 binding.^46^ Hence, we assessed the effect of UVC radiation on pure preparations of tyrosine, as well as tryptophan. Both 227 and 266 nm radiation yielded a dose-dependent loss of aromatic ring conformation (SI Figure S10), broadening of UV-absorption peaks, as well as the loss of sharp aromatic vibration peaks in the Raman spectrum, which were replaced by broad C–C bond vibrations ∼930 cm^–1^ (SI Figure S9B). Together, this suggests, as expected, that UVC irradiation causes degradation of the aromatic rings in Tyr and Trp.^47^

### 227 and 266 nm-Induced Protein Unfolding Have Similar Molecular Mechanisms

In order to identify, in an unbiased manner, differences in mechanism between 227 and 266 nm radiation we performed principal component analysis (PCA) on the Raman spectra and selected components representing UVC dose-dependent changes (Figure 5G, 5H). Interestingly, both loadings spectra show that the same features, albeit with differing coefficients, are responsible for UVC dose-dependent changes (Figure 5I). The same result was observed for PCA of the normalized Amide I region alone (SI Figure S11). This suggests that both wavelengths induce damage by similar mechanisms. Key peaks in the loadings spectra that were associated with the native protein include: 933 and 1296 cm^–1^ (α-helix), 995 and 1243 cm^–1^ (β-sheet), and 758, 869, 1010, 1216, 1576, and 1615 cm^–1^ (aromatics). Key peaks in the loadings spectra that were associated with UVC irradiation included 983, 1268, 1665, and 1685 cm^–1^ (nonregular structure).^48^ We next included all spectra for both 227 and 266 nm irradiated rSARS-CoV-2 S in the same PCA. Across PC1 (dose-dependence of UVC radiation) 227 nm irradiated rSARS-CoV-2 S spectra have larger scores than 266 nm (SI Figure S11). From the above analysis we conclude that while both wavelengths have a similar mechanism of action, the effect of 227 nm radiation is stronger, particularly on secondary structure. It is possible that the damage to aromatic residues and disulfide linkage, which are similarly effected by both 227 and 266 nm wavelengths are the main contributors to loss of S proteins binding ability and may explain the large loss in binding capacity observed at 100 mJ (Figure 3).

### UVC Irradiation Does Not Cause sSARS-CoV-2 S Glycan Loss

The S protein is known to be decorated with 22 N-glycans per subunit,^24,49^ with glycosylation contributing to the stabilization of the RBD conformation,^50^ We observed changes in vibrations that relate to the glycosylation status of rSARS-CoV-2 S, but many overlap with those originating from protein bonds.^51^ However, we tentatively assigned the dose-dependent decrease in the vibration ∼1465 cm^–1^ to glycan CH_2_ deformation and their degradation (SI Figure S12). SDS-PAGE analysis showed that UVC irradiation did not reduce glycosylation, but instead reduced Coomassie binding due to protein degradation.

## Conclusions

Here we have demonstrated the inactivation of SARS-CoV-2 by 266 nm UVC, which matches closely with the absorption spectra of RNA and aromatic amino acids. 266 nm light caused RNA damage at low powers, and we show in detail the mechanisms by which 266 nm irradiation also damages conformation in recombinant SARS-CoV-2 spike protein through the cleavage of disulfide bonds and degradation of aromatic amino acids, reducing its ability to bind hACE2. Importantly we also investigated the effectiveness of 227 nm which is well matched to protein backbone absorption. It was more effective at generating protein damage through oxidation and the unfolding of secondary structure. Reduced rSARS-CoV-2 S binding of hACE2 was observed as a result of UVC exposure that correlated well with changes in hydrophobicity and conformation. As expected, 227 nm radiation was less effective at inducing RNA damage. Notably SARS-CoV-2 has among the largest of genomes for RNA viruses,^52^ making it especially sensitive to genomic damage. Therefore, the role of protein damage may be of even greater importance against other pathogens with smaller genomes. We therefore suggest that both wavelengths, leveraging dual inactivation mechanisms, could be more effective in preventing infectivity of viruses.

The results of inactivation studies with SARS-CoV-2 virus indicate a dose dependent inactivation but at doses that are >11.7 times higher than anticipated by our RNA damage assay, and 5–10 times lower than required to prevent recombinant spike protein binding hACE2. In addressing this difference we note that the ability of host cell ribosomes to process damaged RNA may differ from that of reverse transcriptase used in our assay. In addition, the recombinant spike protein used in the binding assays is more stabilized likely due to additional interactions compared to the wild-type spike protein. Further, the binding assay used does not detect the ability of the S protein to facilitate cell fusion, which likely also declines along with loss of hACE2 binding, or of damage to other viral proteins that may contribute to inactivation. Therefore, the overall effect of UVC protein damage on viral inactivation is likely to be underestimated.

Nonetheless we suggest a hierarchy of sensitivity to UVC irradiation with genomic damage preceding viral protein deactivation. We also note that for viruses with smaller genomes the relative contribution of RNA damage will decline, potentially increasing the importance of proteins as targets for UVC inactivation. How the structures of the both the genome and expressed proteins impact this hierarchy warrants further investigation. In summary we reveal dosages and mechanisms of SARS-CoV-2 inactivation that will also be applicable to other pathogens. Our work provides fundamental evidence that helps understand molecular targets of UVC wavelengths and dosage requirements for high throughput disinfection systems and devices to prevent the transmission and spread of airborne diseases, including Covid-19.

## Supporting Information Available

Materials and methods covering UV laser, experimental setups, methodology details on fluorescence, AFM, Raman, and SPR measurements; details and assay descriptions; expression, purification, and various steps involved to obtain SARS-CoV-2 spike and hACE2 proteins; Table S1 on SPR parameters; SI Figures S1–S14: photographs of assay results, modeling results, additional description of laser doses and results from dose-dependence studies with AFM, fluorescence (Bis-ANS), UV–vis, and Raman spectroscopy on the spike protein, amino acids, and analysis using PCA along with glycosylation results with gel electrophoresis; and schematic of setup and absorbance data of media at 227 and 266 nm (PDF)
