# Supplementary material for: Mechanisms of SARS-CoV-2 Inactivation Using UVC Laser Radiation
Source: ACS Photonics. 2023 Dec 26;11(1):42–52. doi: 10.1021/acsphotonics.3c00828 (PMC10797618; doi:10.1021/acsphotonics.3c00828)
Supplement: Supplementary file 1 — ph3c00828_si_001.pdf [file ph3c00828_si_001.pdf]

## Supplementary Information

# Mechanisms of SARS-CoV-2 Inactivation using UVC Laser Radiation

George Devitt[1,2,3#], Peter B. Johnson[1,3#], Niall Hanrahan[1,3#], Simon I.R. Lane[1,3#], Magdalena C. Vidale[2], Bhavwanti Sheth[2], Joel D. Allen[2], Maria V. Humbert[4,5], Cosma M. Spalluto[4,7], Rodolphe C. Hervé[2], Karl Staples[4,6,7], Jonathan J. West[3,8], Robert Forster[9], Nullin Divecha[2], Christopher J McCormick[4], Max Crispin[2], Nils Hempler[9], Graeme P. A. Malcolm[9] and Sumeet Mahajan[1,3,10*]

[1]School of Chemistry, Faculty of Engineering and Physical Sciences, University of Southampton, Highfield, Southampton SO17 1BJ, UK.
[2]School of Biological Sciences, Faculty of Environmental and Life Sciences, University of Southampton, Highfield, Southampton SO17 1BJ, UK.
[3]Institute for Life Sciences, University of Southampton, Highfield, Southampton SO17 1BJ, UK.
[4]Clinical and Experimental Sciences, Faculty of Medicine, University of Southampton, Sir Henry Wellcome Laboratories, University Hospital Southampton, Southampton SO16 6YD, UK.
[5]University of Cambridge, MRC Toxicology Unit, Cambridge, CB2 1QR, UK.
[6]Wessex Investigational Sciences Hub, University of Southampton Faculty of Medicine, Southampton General Hospital, Southampton SO16 6YD, UK.
[7]Southampton NIHR Biomedical Research Centre, Southampton General Hospital, Southampton SO16 6YD, UK.
[8]Cancer Sciences, Faculty of Medicine, University of Southampton, Southampton SO16 6YD, UK.
[9]M Squared Lasers Ltd, 1 Kelvin Campus, West of Scotland Science Park, Glasgow, G20 0SP, UK.
[10]Department of Biotechnology, Holsetgata 22, N-2317 Hamar, Inland Norway University of Applied Sciences.

# Contents

I. **Materials and Methods**
II. **Supplementary Tables: Table 1**
III. **Supplementary Figures: Figures 1-14**

## I. Materials and Methods

### UV lasers and illumination through liquid light guide

The light source used for the experiments was based on a high-brightness, single-frequency, continuous-wave, solid-state laser operating at 532nm with a maximum output power of 20W (Equinox, M2 Lasers, Glasgow, UK). The 532nm light was doubled in frequency to 266nm by using a commercial cavity-enhanced second harmonic generation module (ECD-X-Q, M2 Lasers, Glasgow, UK). For the purpose of the experiments, the output power of the 532nm laser was limited to 2W, resulting in up to 468mW of 266nm light.

For the 227nm light, the same 532nm solid-state laser was used, however, the output was first used to pump a commercially available, highly frequency agile titanium sapphire laser (SolsTiS, M2 Lasers, Glasgow) that provided up to 6W of output power.

The laser and nonlinear conversion module were mounted on an optical breadboard for mobility, which was placed on either an optical bench during initial laboratory testing, or on a compact mobile trolley for working in a Containment Level 3 laboratory. The UV laser output passed through an optical shutter (SH1/M, Thorlabs) and was coupled into a 5mm core liquid light guide (LLG) (LLG5-4T, Thorlabs, UK) using two aluminium mirrors (PF10-03-G01, Thorlabs). The flexible LLG delivered high-power UV to the sample surface, and UV emission from the LLG was a uniform circle which diverged slowly. At 20mm from the sample surface, the UV illumination area covered a 23mm diameter circle. Exposure times were controlled using the electronic shutter (**Supplementary Figure S13**).

$$\theta = \sin^{-1}(\frac{NA}{n})$$

$$d = 5 + 2s\tan(\theta)$$

d = Spot size; s = Vertical distance to sample surface; NA = 0.42; A = LLG aperture size (5 mm)

### UV illumination of liquid samples

A liquid sample (1-15µL) was pipetted onto the smooth upper surface of the central PMMA pedestal, and a quartz slide (UQG optics) was placed on top, providing a uniform thickness across the sample for UV exposure. The sample was illuminated with UV from above, through the quartz slide. After exposure, samples were recovered from the quartz and PMMA surfaces. More than 90% of the sample was typically recovered. UV transmission through the quartz slide was >92% at 266nm, and >86% at 227nm. Quartz slides were stored in 70% ethanol when

not in use, and the quartz slides and sample chambers were cleaned with 70% ethanol between each sample irradiation.

**UV transmission through liquids**

Culture media (2mL) was placed in a quartz cuvette, aligned perpendicular to the beam propagation direction, and UV power transmission was measured through the filled cuvette with a power meter (30A-BB-18, Newport). 1mL of media was removed and replaced with 1mL DI water, and UV power transmission was measured again. This was repeated for sequential 2x dilutions of media with DI water to a $2^{-15}$ concentration. This was performed for DMEM supplemented with 0%, 4% or 10% FBS (**Supplementary Figure S14)** yielding molar extinction coefficients ($mol^{-1}.cm^{3}$).$cm^{-1}$ for 266nm: 10%, 7.9; 4%, 4.8; 0%, 2.8 and 227nm: 10%, 48.3; 4%, 15.8; 0%, 7.4. The transmitted powers through the respective media used in each experiment was used for dosage calculations (also see Supplementary Figure S4).

**SARS-CoV-2 Virus and associated cell lines**

Human coronavirus SARS-CoV-2 (BetaCoV/Australia/VIC01/2020) and a kidney cell line (Vero E6) were obtained from Public Health England (PHE), UK. Uninfected cells were maintained in DMEM (Invitrogen; cat no 11995065) supplemented with 10% foetal bovine serum (FBS), glutamine and 50u/ml penicillin-streptomycin at 37°C in 5% $CO_2$. To produce working stocks of virus, cells were infected at a multiplicity of infection of 0.005 then maintained for 3 days in DMEM supplemented with 4% foetal bovine serum, 50U/mL penicillin-streptomycin and 25mM HEPES. The resultant cell culture supernatant was clarified by centrifugation at 2,000g for 10 minutes and frozen as aliquots at -80°C.

All handling of live and inactivated SARS-CoV-2 virus was performed in level 3 containment laboratories, with dried samples being handled in a class II microbiological safety cabinet (MSC) and liquid samples in a class III MSC.

**UV inactivation assay for SARS -CoV-2**

25µL microliters of SARS-CoV-2 ($1x10^6$ $pfu.mL^{-1}$) were placed in the centre of alternate wells of a 24-well plate and left to dry under ambient conditions for a period of 3 hours. Laser power from the LLG was set for continuous exposure at specified output power. Dried virus samples were illuminated with UV at 266nm for 1, 3, 10, 30, and 100s, and all exposures were repeated

in triplicate. The samples in the polystyrene well plates were exposed from the top without the lid in place. Each exposed sample was resuspended in 0.5ml serum-free DMEM supplemented with 25mM HEPES (infection medium) for a period of 30 minutes and the samples and their respective dilutions assessed by plaque assay.

**SARS-CoV-2 plaque assay**

Vero E6 cells were seeded at 2.5x$10^5$ cells/well in a 12-well plate and left for a period of 24 hours. Cells were washed once with infection medium and 0.4ml virus-containing supernatants added to each well. After a 1 hour incubation at 37°C in 5% $CO_2$, infectious supernatants were removed and a 1.5mL overlay of 1 x DMEM supplemented with 4% FBS, 25mM HEPES and 0.6% (w/v) cellulose (Sigma; cat no 435244) was added. Plates were incubated at 37°C and 5% $CO_2$ for 72 hours before removing the overlay, fixing with 8% formaldehyde in PBS, and staining with 0.1%(w/v) crystal violet in a solution of 20% (v/v) ethanol.

**RNA damage assay**

MS2 phage ssRNA (Sigmal Aldrich) was resuspended at a concentration of 80ng/uL in nuclease-free water and 15µL drops placed in the lid of a sterile 96-well plate were irradiated with 21mW power using 227nm or 266nm lasers, for 0, 1, 3, 10, 30 or 100s. 10 µL of RNA solution was recovered (800ng RNA) and reverse transcribed using a Precision Nanoscript2 RT kit (Primer Design Ltd) using a first strand synthesis primer (CCAAATCGGGAGAATCCCGGGTCC). 10ng of the equivalent cDNA generated was then subjected to qPCR using a qPCRBio SyGreen Kit (PCR Biosystems Ltd) using two primer pairs situated 767-925 bases (ATCCGCTCGCACTACGGAAT, ATGCCTATGGTTCCGGCGTT) or 2087-2231 bases (AGAGCCCTCAACCGGAGTTT, TAAGCCTGTGAGCGCGAGTT) from the 5' end of the first strand cDNA. 40 cycles of denaturation at 95°C, 5s; and annealing/extension at 62°C, 30s; were performed using StepOne Plus machine (Applied BioSystems). Each reaction was carried out in duplicate and deltaCt values were calculated and normalised against non-irradiated controls. Curve fitting analysis was performed using prism8 (Graphpad Software LLC). 99.9% inactivation doses were calculated from the fitted curve.

**UV dose-dependent lesion scaling with ssRNA genome length**

Considering light interacting with RNA genomes, replication inhibition by UVC lesion effects were modelled using Poisson statistics involving a linear proportionality relating to the genome

length. For predicting wavelength dependent doses required to inactivate 99.9% of a virus with a given ssRNA genome (e.g. Influenza A, 13,588nt; and SARS-CoV-2, 30,000nt). The probability model was normalized using the experimental replication inhibition values obtained with 21mJ/cm$^2$ UV doses interacting with the MS2 2,231 base fragment.

**Expression and purification of trimeric recombinant SARS-CoV-2 spike protein**

To express the prefusion S ectodomain, a gene encoding residues 1−1208 of SARS-CoV-2 S (GenBank: MN908947) with proline substitutions at residues 986 and 987, a "GSAS" substitution at the furin cleavage site (residues 682–685), a C-terminal T4 fibritin trimerization motif, an HRV3C protease cleavage site, a TwinStrepTag and an 8XHisTag was synthesized and cloned into the mammalian expression vector pαH. Expression plasmid encoding SARS-CoV-2 S glycoprotein 6 was transiently transfected into Human Embryonic Kidney (HEK) 293F cells. Cells were maintained at a density of 0.2-3x10$^6$ cells per mL at 37°C, 8% $CO_2$ and 125rpm shaking in FreeStyle 293F media (Fisher Scientific). Prior to transfection two solutions containing 25mL Opti-MEM (Fisher Scientific) medium were prepared. Plasmid DNA was added to one to give a final concentration after transfection of 310µg/L. Polyethylenimine (PEI) max reagent (1mg/mL, pH 7) was added to the second solution to give a ratio of 3:1 PEI max: plasmid DNA. The two solutions were combined and incubated for 30 minutes at room temperature. Cells were transfected at a density of 1x10$^6$ cells per ml and incubated for 7 days at 37°C with 8% $CO_2$ and 125 rpm shaking.

After harvesting, the cells were spun down at 4000rpm for 30 minutes and the supernatant applied to a 500mL Stericup-HV sterile vacuum filtration system (Merck) with a pore size of 0.22µm. The supernatant containing SARS-CoV-2 S protein was purified using 5mL HisTrap FF column connected to an Akta Pure system (GE Healthcare). Prior to loading the sample, the column was washed with 10 column volumes of washing buffer (50mM $Na_2PO_4$, 300mM NaCl) at pH 7. The sample was loaded onto the column at a speed of 2mL/min. The column was washed with washing buffer (10 column volumes) containing 50mM imidazole and eluted in 3 column volumes of elution buffer (300mM imidazole in washing buffer). The elution was concentrated by a Vivaspin column (100kDa cut-off) to a volume of 1mL and buffer exchanged to phosphate buffered saline (PBS).

The Superdex 200 16 600 column was washed with PBS at a rate of 1mL/min. After 2 hours, 1mL of the nickel affinity purified material was injected into the column. Fractions

separated by SEC were pooled according to their corresponding peaks on the Size Exclusion chromatograms. The target fraction was concentrated in 100kDa vivaspin (GE healthcare) tubes to ~1mL.

### Expression and purification of hACE2

FreeStyle293F cells (Thermo Fisher) were transfected with polyethyleneimine and a plasmid encoding residues 1-615 of human ACE2 with a C-terminal HRV3C protease cleavage site, a TwinStrep Tag and an 8XHisTag. This construct is identical to full length ACE2 except is truncated at position 626. This protein was expressed and purified identically as for the hSARS-CoV2 S glycoprotein, with the exception of a smaller Vivaspin cutoff being used for buffer exchanging.

### His-Tag removal of hACE2

Following purification, the His-Tag was removed from hACE2 using HRV3C protease cleavage (Thermo Fisher). Digestion was performed at a ratio of 1:20 HRV3C protease: ACE2 in 1x HRV3C reaction buffer (Thermo Fisher) and incubated at 4°C overnight. To remove the HRV3C and uncleaved hACE2 nickel affinity chromatography was performed, except the flow through was collected rather than the elution.

### Absorbance Spectroscopy

Absorbance spectroscopy was performed using a NanoDrop 2000 spectrometer. Spectra were collected between 220nm and 340nm using a 2µl sample, all spectra were collected immediately after UV irradiation to minimise contributions from aggregation. Data was extracted from an average of 3 repeat readings, no further processing was performed. Erroneous data was rejected if spectra were flat or unusually noisy, typically due to the presence of an air bubble during data collection. Nonlinear curve fit analysis was performed using Origin 2020.

### Tryptophan fluorescence

Tryptophan fluorescence was performed using a Cary Eclipse fluorescence spectrophotometer, excitation was set to 280nm and an emission collected between 300nm and 500nm, excitation

and emission slit widths were set to 5nm. Protein concentration was 9µg/mL in all cases. Displayed spectra are blank subtracted averages of 3 repeat readings and have undergone nearest neighbour averaging with a 5-point window.

**Bis-ANS fluorescence**

Bis-ANS (Sigma) was dissolved in PBS and mixed with protein samples after irradiation to a final concentration of 10µM dye and 0.4mg/ml protein. Solutions were transferred to 96-well plates (Costar) and fluorescence was measured using an excitation filter of 450nm and an emission filter of 490nm. Each experiment was performed using two technical duplicates. The fluorescence of each equivalent blank solution (buffer + dye) was subtracted from each experimental readout and fluorescence was normalized as fold change over native rSARS-CoV-2 S protein fluorescence.

**Atomic force microscopy (AFM)**

20µL of 2µg/mL protein was added to a freshly cleaved 10mm mica disc (Agar Scientific) and incubated at room temperature for 2 min. The protein solution was then washed with 0.22µM filtered, double distilled $H_2O$ three times before drying in air. Samples were imaged using a Digital Instruments Multimode IV AFM system operated in tapping mode. Aluminum-coated, noncontact/tapping mode probes with a resonance frequency of 320kHz and force constant of 42N/m were used for all images (Nanoworld, POINTPROBE NHCR). Probes were autotuned using Nanoscope III 5.12r3 software before use. Images shown are representative of the sample. Two 5µm images were taken at random points on the sample per experiment with a scan rate of 1–2Hz and 512 samples per line/512 lines per image. Height analysis was performed using the particle analysis feature in WSxM Beta[1] using 10 bins with a range of 0-50nm.

**Raman spectroscopy**

A Renishaw InVia microscope system was used for Raman spectroscopy. Quartz coverslips were coated in trichloro(1H,1H,2H,2H-perfluoroocytl)silane (Sigma) by chemical vapor deposition. A silane atmosphere was created by a 30 min desiccation using a small volume of trichloro(1H,1H,2H,2H-perfluoroocytl)silane in a reaction chamber. Quartz coverslip surfaces were activated by oxygen plasma treatment before incubation in the silane atmosphere to create a silane monolayer on the coverslip surface for 2h. For Drop-deposition Raman spectroscopy (DDRS), 0.5µL of each protein sample was first dried onto a quartz coverslip for 12 min under

vacuum. Spectra were then collected from the "coffee ring" of each drop, where proteins are found in the absence of bulk salt.[2-4] The samples were excited using a 785nm laser focused through a Leica 50× (0.75 NA) short working distance objective for DDRS. Data was obtained and parameters were set using Renishaw WIRE4.1 software, WIRE was also used for cosmic ray removal. Spectra were collected in the fingerprint region (614–1722cm$^{-1}$). The Raman system was calibrated to the 520cm$^{-1}$ reference peak of silicon prior to each experiment.

Spectra were acquired for 5s with 6 acquisitions in different locations on the coffee ring per experiment. A total of 15 spectra were collected for native rSARS-CoV-2 S and 11 spectra were collected for each UVC condition. Erroneous spectra were rejected with unusual background fluorescence that could not be removed using polynomial subtraction. Presented Spectra represent the class means of all remaining spectra. Preprocessing and Principal Component Analysis (PCA) was performed using the IRootLab plugin (0.15.07.09-v) for MATLAB R2015a.[5] All spectra were background-subtracted using blank quartz spectra and were smoothed using the wavelet denoising function. A fifth-order polynomial was used to remove fluorescence and the ends of each spectra were anchored to the axis using the rubberband-like function. Spectral intensity normalization was applied using vector normalisation or using a specific band (each mentioned within captions). Trained-mean centering was then applied to the spectra before PCA with a maximum of ten principle components. Amide I second derivative analysis was performed on class mean spectra using WIRE4.1 software.

**Surface plasmon resonance (SPR)**

rSARS-COV-2 S and hACE2 proteins were buffer exchanged in HBS P+ buffer (Cytiva/GE Healthcare). All analysis was performed using a Biacore T200 (Cytiva/GE Healthcare). After removing metallic contaminants via a pulse of EDTA (350 mM) for 1 min at a flow rate of 30µL/min, the chip was loaded with $Ni_2$+by injecting $NiCl_2$ for 1 min at a flow rate of 10µL/min. SARS-CoV2 S protein (50,000ng/mL) was injected at 10µL/min for 240s. Control channels received neither trimer nor $NiCl_2$. Control cycles were performed by flowing the analyte over $Ni_2$+-loaded NTA in the absence of trimer; there were no indications of non-specific binding. The analyte was injected into the trimer sample and control channels at a flow rate of 50µL/min. Serial dilutions ranging from 200nM to 3.125nM were performed in triplicate along with HBS P+ buffer only as a control. Association was recorded for 300s and dissociation for 600s. After each cycle of interaction, the NTA-chip surface was regenerated with a pulse of EDTA (350mM) for 1 min at a flow rate of 30µL/min. A high flow rate of analyte solution

(50µL/min) was used to minimize mass-transport limitation. The resulting data were fit to a 1:1 binding model using Biacore Evaluation Software (GE Healthcare) and these fitted curves were used to calculate KD.

**Custom sample holders for UV illumination**

Sample holders were created from PMMA, a microscope slide, and a quartz slide for illumination of a known liquid sample thickness. PMMA (3mm, Techsoft) was cut into 10mm squares using a laser cutter. Further pieces of PMMA were laser etched to remove a depth of 150µm, and then also cut to 10mm squares. Glass slides were also lightly etched to provide a high surface area for PMMA binding. The etched PMMA pedestal was attached to the centre of a microscope slide with epoxy resin, with the etched surface in contact with the slide. Two 3mm thick PMMA pedestals were similarly attached either side of the central pedestal, resulting in the central pedestal being 150µm lower than the surrounding supports. Final sample thickness was confirmed using digital callipers to be 150±5µm. Samples were placed on the central pedestal, and the quartz glass placed over, resting on the outer pedestals.

## II. Supplementary Table

**Supplementary Table 1: Parameters for SPR binding of SARS-CoV2 and hACE2**

| | KD (nM) | Rmax (RU) | Chi² (RU²) |
|---|---|---|---|
| Untreated | 53.93 | 377.4 | 26.7 |

| 227nm | KD (nM) | Rmax (RU) | Chi² (RU²) |
|---|---|---|---|
| 2200 | n.d. | 7.931 | 2.67 |
| 600 | 85.96 | 51.31 | 1.72 |
| 100 | 59.35 | 129.1 | 14.3 |

| 266nm | KD (nM) | Rmax (RU) | Chi² (RU²) |
|---|---|---|---|
| 2200 | n.d. | 23.73 | 33.2 |
| 600 | 69.38 | 143.3 | 6.32 |
| 100 | 62.05 | 158.1 | 6.63 |

## III. Supplementary Figures

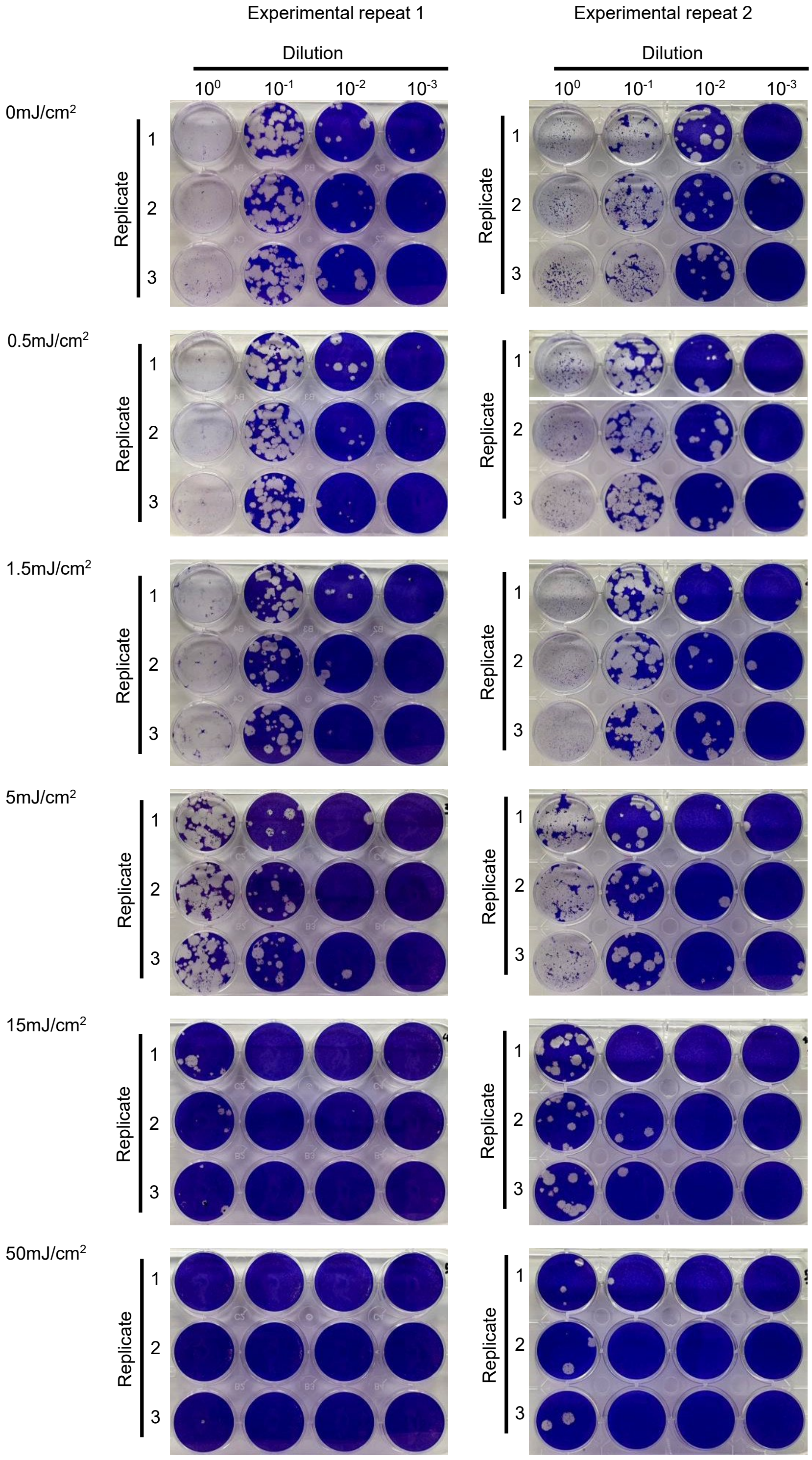


**Supplementary Figure 1. Plaque assay for SARS-CoV-2 virus inactivation after UV irradiation.** SARS-CoV-2 virus was irradiated with 266nm light, with laser power density of 0.5mW/cm$^2$ and exposure times of 1, 3, 10, 30 and 100s, repeated in triplicate. Cell monolayers were stained with crystal violet and fixed with PFA. Images show that with increasing UV dose, fewer plaques formed on cell monolayers infected with UV-irradiated virus stock. 99.9% of virus was inactivated with a dose of 50mJ/cm$^2$.

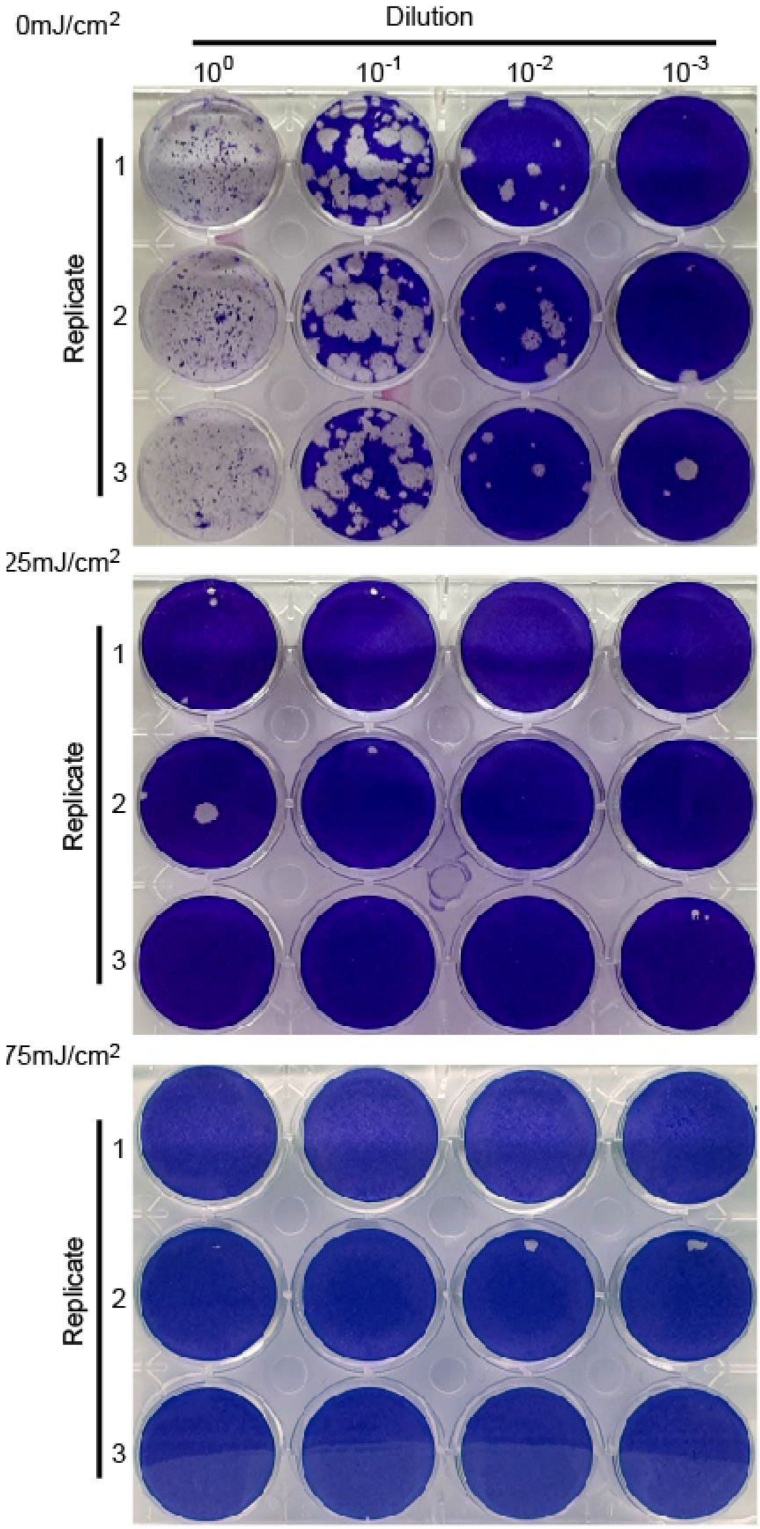


**Supplementary Figure 2. Plaque assay for SARS-C0V-2 virus inactivation after high dose UVC irradiation.** SARS-CoV-2 virus was irradiated with UVC at 266nm, with laser power density of 25mW/cm$^2$ and exposure times of 0, 1 3, 10, 30 and 100s, repeated in triplicate. Cell monolayers were stained with crystal violet and fixed with PFA. Images show a single plaque formed for only one of the three repeats at the lowest UVC dose (25mJ/cm$^2$), and no plaques were observed for higher doses (10, 30 and 100s not shown), indicating complete inactivation of the virus.

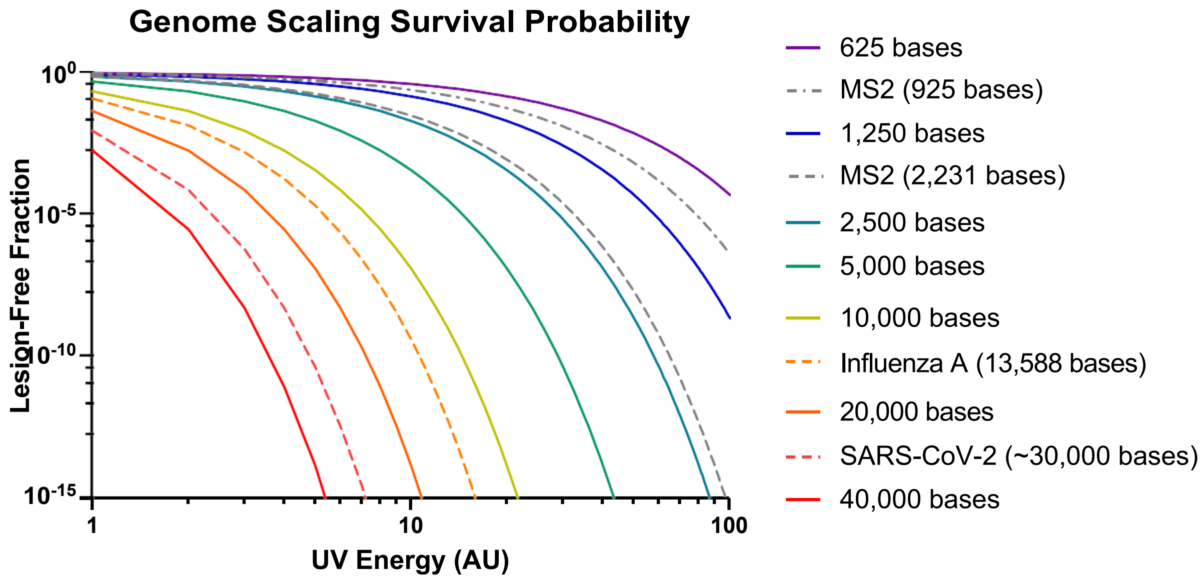


**Supplementary Figure 3. Theoretical genome scaling analysis.** The proportion of RNA that remains lesion free plotted against Energy, for various sized genomes. The probability model was normalized using the experimental replication inhibition values obtained with 21mJ/cm$^2$ UV doses interacting with the ~2.3kb MS2 fragment.

| Time (s) | Power (mW) | Dose (mJ) | Dose classification |
|---|---|---|---|
| 100 | 0 | 0 | |
| 100 | 1 | 100 | *Low* |
| 100 | 3 | 300 | |
| 100 | 6 | 600 | *Medium* |
| 100 | 10 | 1000 | |
| 100 | 22 | 2200 | *High* |
| 100 | 30 | 3000 | |

**Supplementary Figure 4: UVC doses used to irradiate rSARS-CoV-2 S protein.** All doses shown were used for UV-vis absorption spectroscopy. The power indicated is the transmitted power rather than the irradiated power. Highlighted doses were used for experiments in figures 3, 4 and 5.

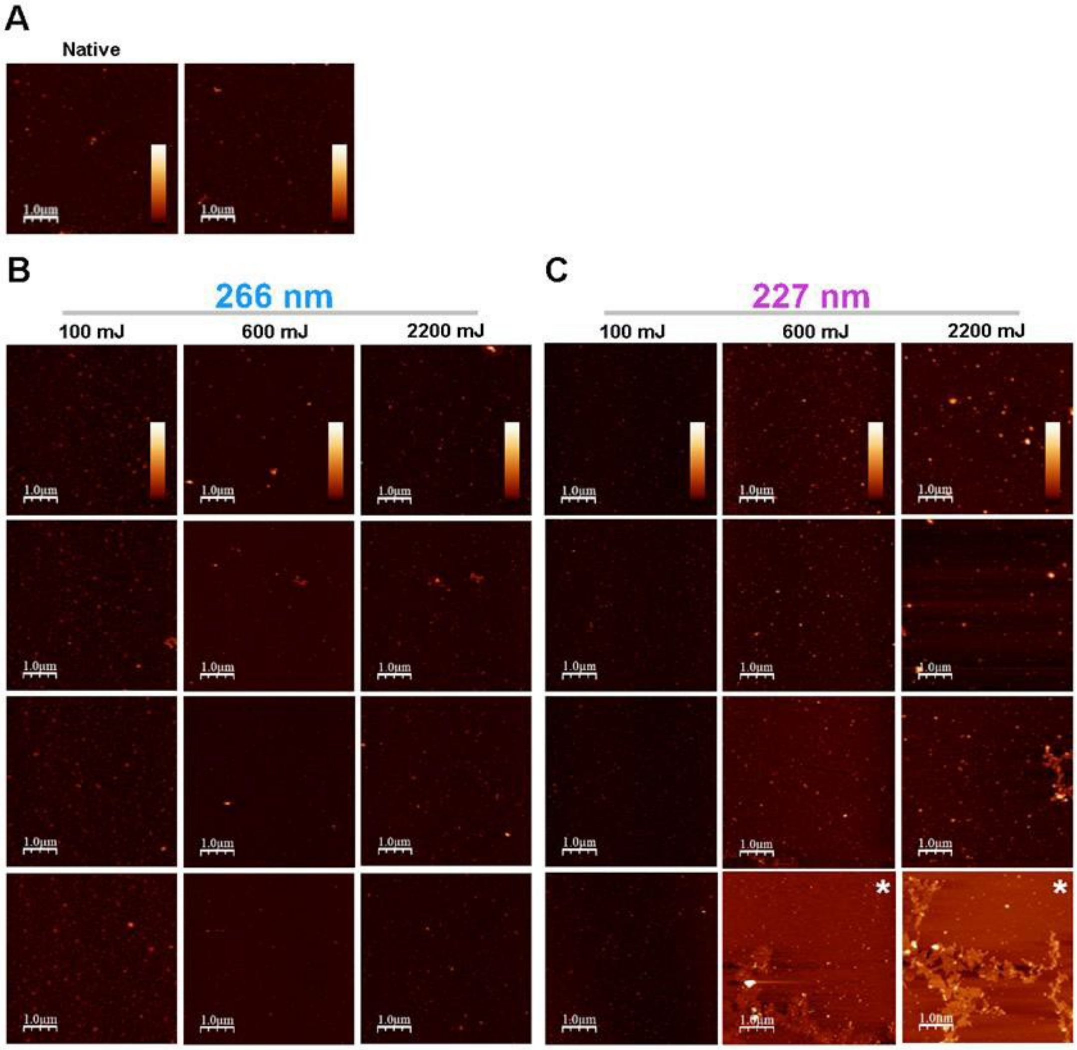


**Supplementary Figure 5. AFM images of SARS-CoV-2 S used for particle height analysis.** AFM images used for particle height analysis shown in Figure 3. AFM images of SARS-CoV-2 S bound to a mica surface in the native from (A), or after UVC irradiation using 266nm light (B) or 227nm light (C). Images are 5µm x 5µm, scale bar is equal to 1µm and z-scale is equal to 0-30nm. Images labelled with * were not used for height analysis as large aggregated structures prevented correct image flattening resulting in incorrect Z-values.

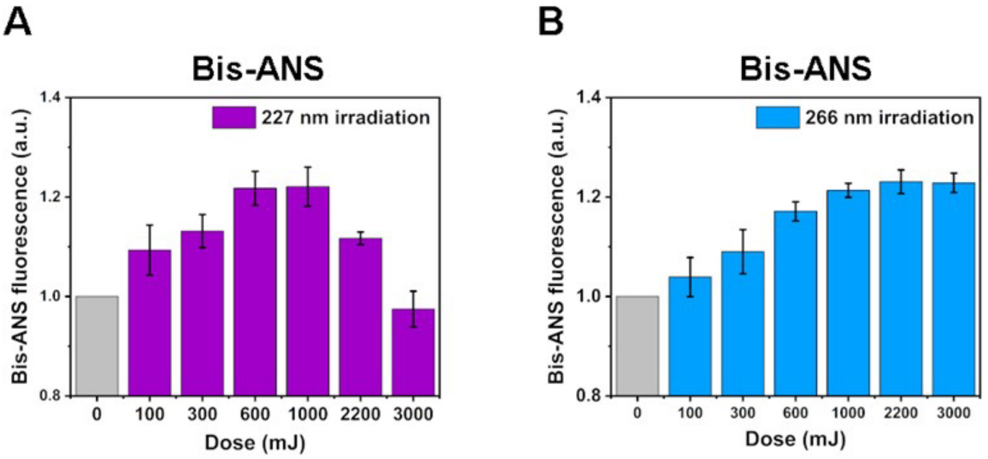


**Supplementary Figure 6. Dose-dependent changes in BSA surface hydrophobicity in response to UVC irradiation follow same pattern as for rSARS-CoV-2 S.** Changes in Bis-ANS binding, measured by fluorescence emission at 490nm, induced by 227nm radiation (A) and 266nm radiation (B). n=2, the plotted data represent the average and SD from four fluorescence readings.

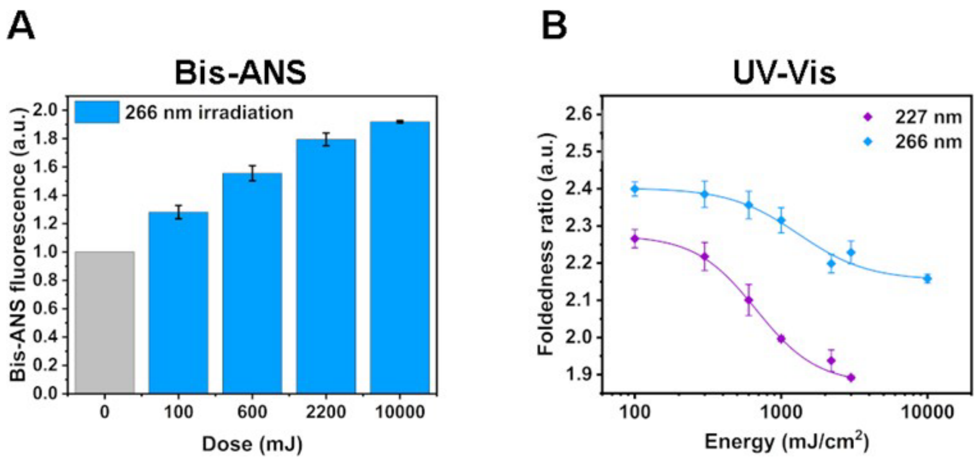


**Supplementary Figure 7. 10J 266nm dose does not induce the same conformational effects in SARS-CoV-2 S as lower doses 227nm radiation.** A) Changes in Bis-ANS binding measured by fluorescence emission at 490nm induced by 266nm radiation. B) Dose-dependence of rSARS-CoV-2 spike protein foldedness ratio to UVC radiation determined by UV-vis absorption spectroscopy. Foldedness ratio = A280nm/A275nm + A280nm/A258nm. n=1. The plotted data for 10000mJ represents the average and SD from 2 fluorescence/absorbance readings, all other plotted data represent the average and SD from 4 fluorescence/absorption readings (n=2).

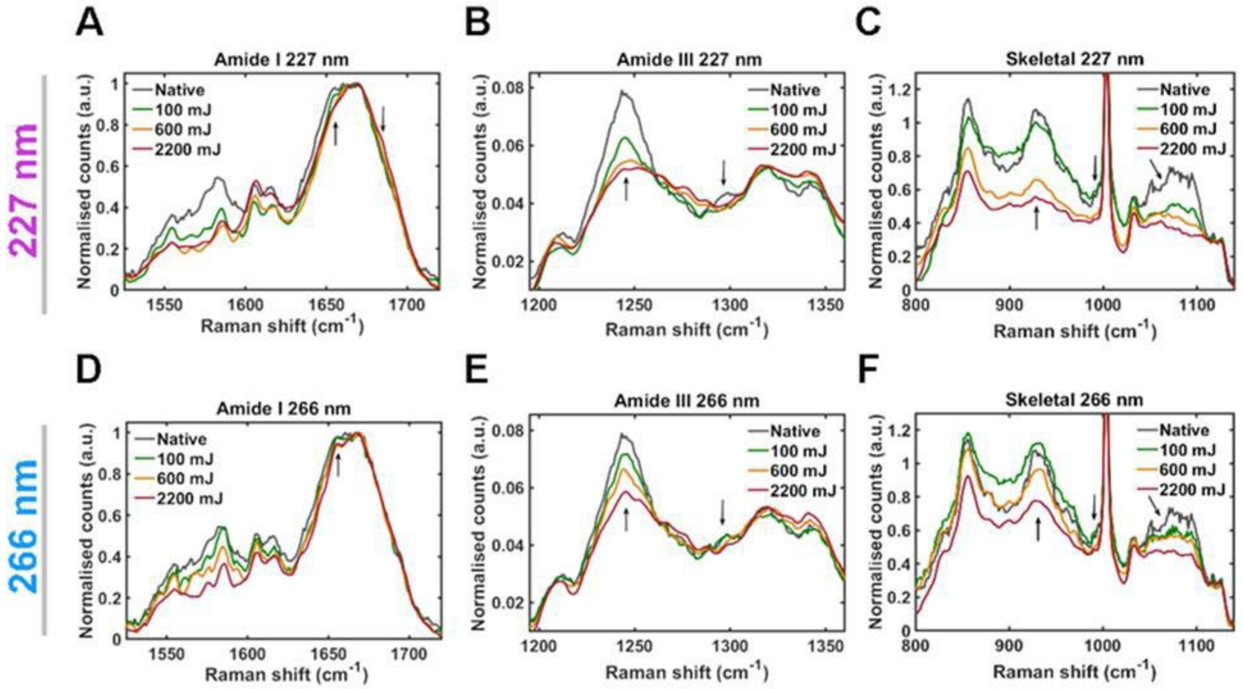


**Supplementary Figure 8. UVC dose-dependence of rSARS-CoV-2 spike protein secondary structure determined by Raman spectroscopy.** Changes in Raman spectra induced by 227nm radiation (A-C) or 266nm (D-F). A, D) Amide I spectra from 1525-1722cm$^{-1}$. Arrows indicate points of variation due to change of UVC dose. Upwards arrows indicate α-helix vibration, downwards arrow indicates nonregular structure vibration. B, E) Amide III spectra from 1195-1360cm$^{-1}$. Upwards arrow indicates β-sheet and mannose vibrations, downwards arrow indicates α-helix vibration. C, F) Skeletal region spectra from 800-1140cm$^{-1}$. Upwards arrow indicates α-helix vibration, downwards arrow indicates β-sheet and mannose vibration. n=2, plotted spectra represent the class means of 10-15 spectra per class.

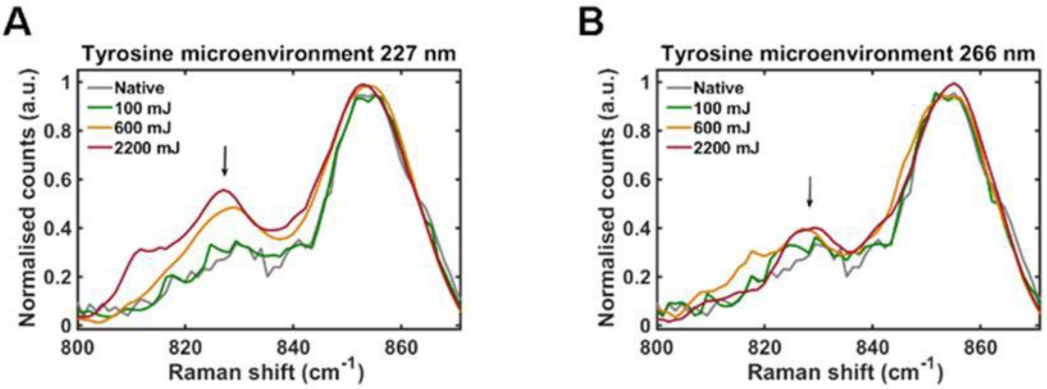


**Supplementary Figure 9. UVC dose-dependence of rSARS-CoV-2 spike protein tyrosine microenvironment determined by Raman spectroscopy.** A) Changes in 830cm$^{-1}$/850cm$^{-1}$ ratio induced by 227nm radiation. B) Changes in 830cm$^{-1}$/850cm$^{-1}$ratio induced by 266nm radiation. Spectra are normalised to ~850cm$^{-1}$ tyrosine peak. Arrows indicate 830cm$^{-1}$ tyrosine peak. n=2, plotted spectra represent the class means of 10-15 spectra per class.

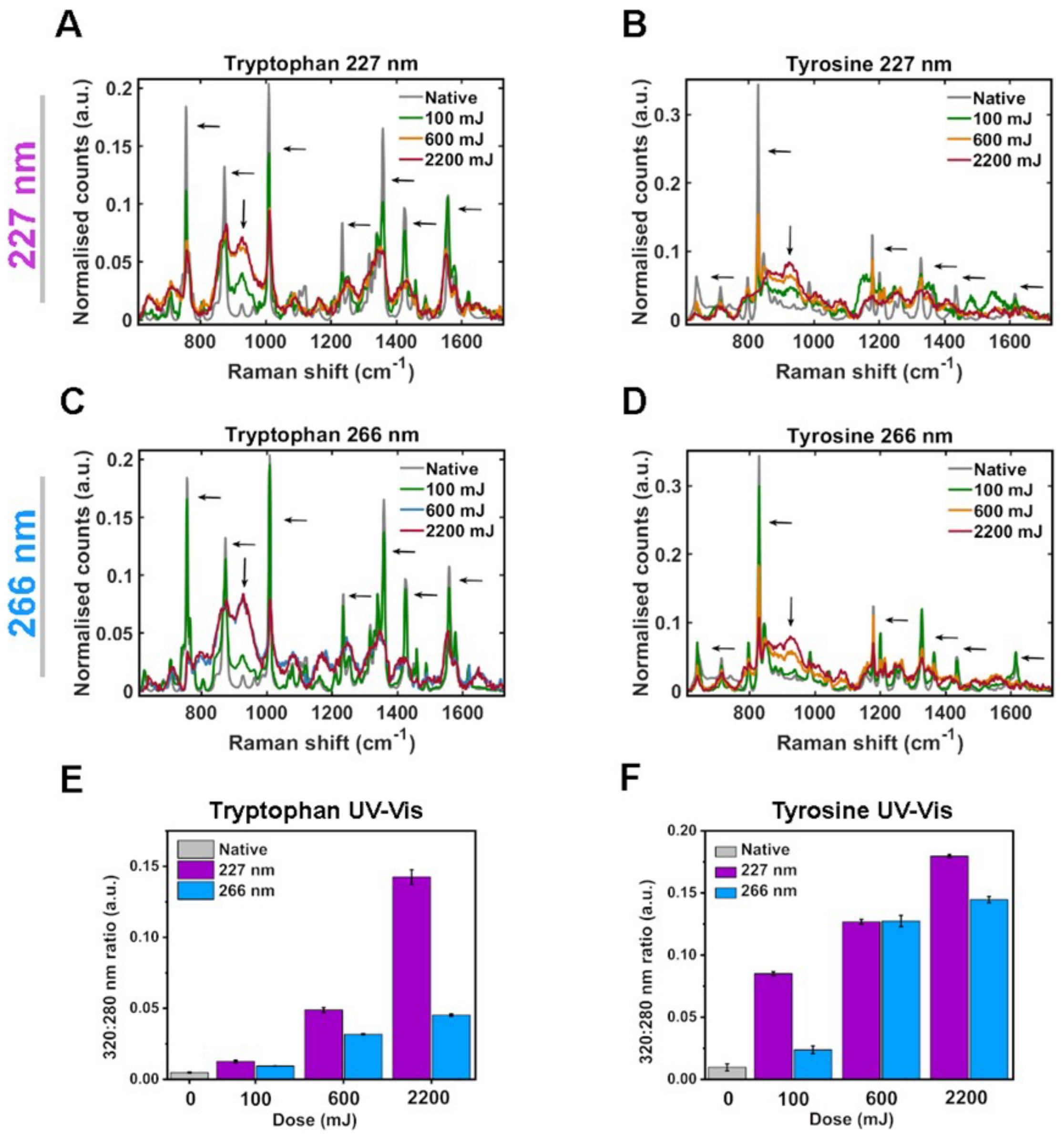


**Supplementary Figure 10. UVC dose-dependence degradation of pure aromatic amino acids.** A-B) Changes in Raman fingerprint induced by 227nm radiation for tryptophan (A) and tyrosine (B). Downwards arrows indicate C-C vibrations and horizontal arrows indicate aromatic vibrations. C-D) Changes in Raman fingerprint induced by 266nm radiation for tryptophan (A) and tyrosine (B). Downwards arrows indicate C-C vibrations and horizontal arrows indicate aromatic vibrations. n=1, plotted spectra represent the class means of 3 spectra per class. E-F) Dose-dependence of tryptophan (E) and tyrosine (F) oxidation ratio to UVC radiation determined by UV-vis absorption spectroscopy. Oxidation ratio = A320/A280nm. n=1, the plotted data represent the average and SD from 3 absorbance readings per class.

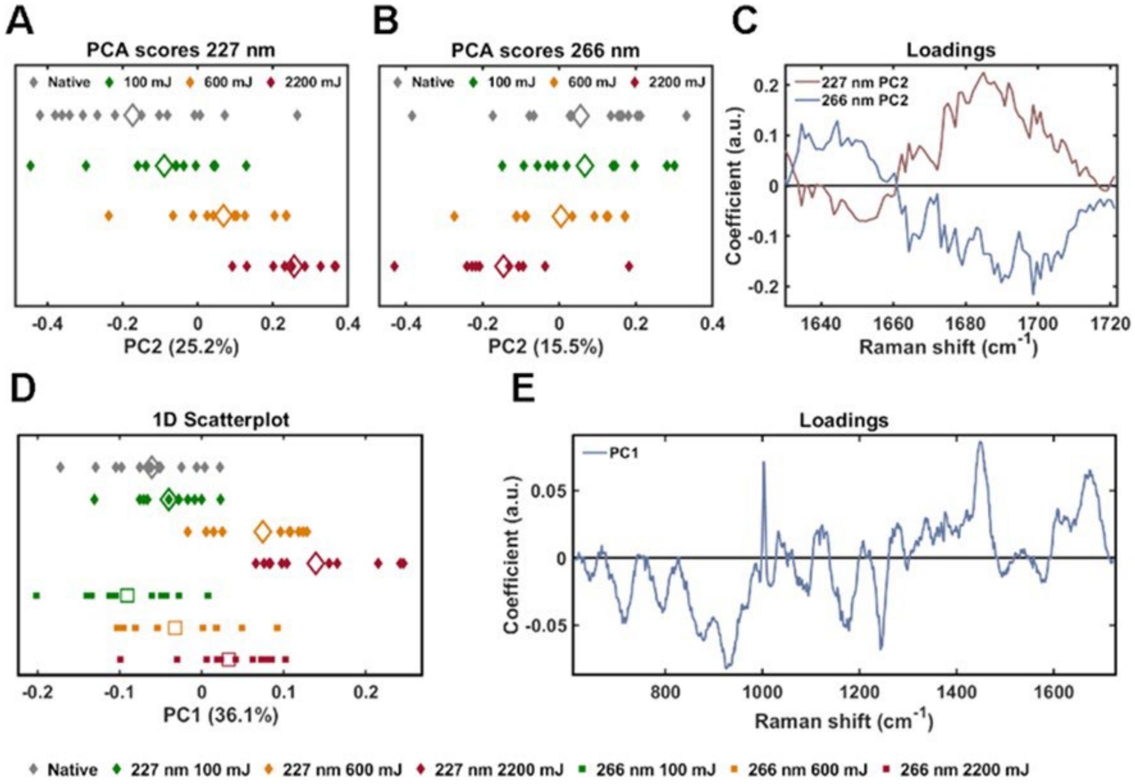


**Supplementary Figure 11. PCA of rSARS-CoV-2 S Raman spectra.** A-B) 1-dimensional principle component analysis (PCA) scores plot of Amide I Raman spectra (1635-1722cm$^{-1}$) for 227nm (A) and 266nm (B) irradiated rSARS-CoV-2 S. Each solid diamond represents the PC score of a single spectrum. Hollow diamonds represent mean score. C) PC loadings spectra representing the spectral variation responsible for the score across the given PC axis. D) 1-dimensional principle component analysis (PCA) scores plot of Raman spectra (622-1722cm$^{-1}$) for 227nm and 266nm irradiated rSARS-CoV-2 S. Each solid diamond/square represents the PC score of a single spectrum. Hollow diamonds represent mean score. E) PC loadings spectra representing the spectral variation responsible for the score across the given PC axis. n=2, PCA performed on 10-15 spectra per class, 10 PCs were retained.

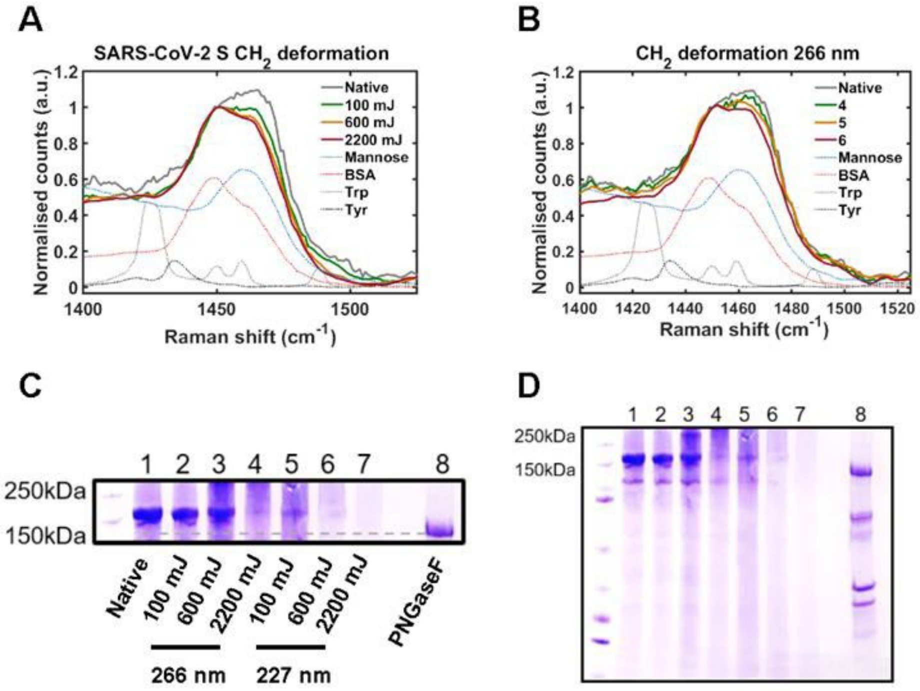


**Supplementary figure 12. UVC irradiation of SARS-CoV-2 does not cause a decrease of glycosylation.** A) Changes in glycan $CH_2$ deformation induced by 227nm radiation and measured by Raman spectroscopy. B) Changes in glycan $CH_2$ deformation induced by 266nm radiation and measured by Raman spectroscopy. n=2, plotted spectra represent the class means of 10-15 spectra per class. C) SDS PAGE analysis of SARS-CoV-2 glycosylation status. D) Full gel of data shown in C. n=1.

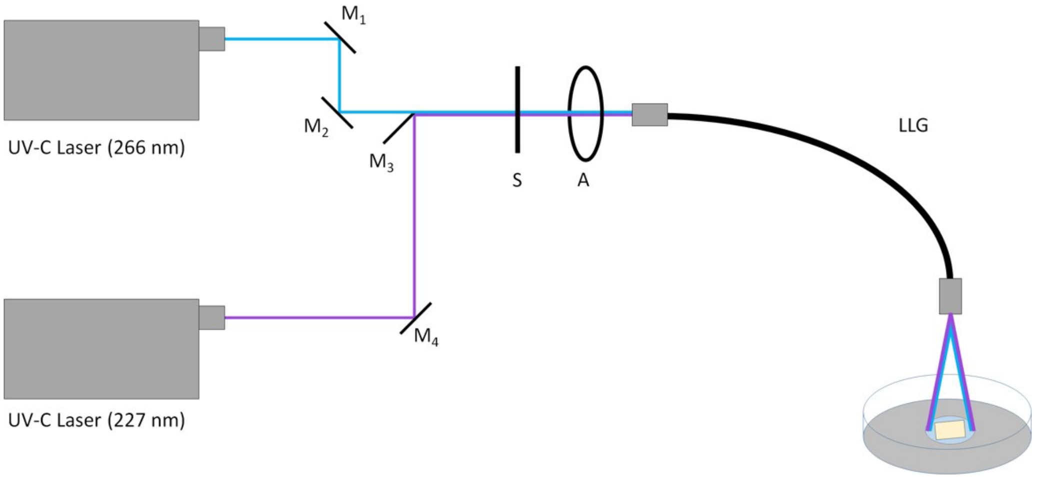


**Supplementary Figure 13. Diagram of dual-laser UV-illumination setup.** Two UVC lasers, emitting at 227nm and 266nm, were coupled through an electronic shutter and adjustable aperture, and then into a liquid light guide (LLG). The large aperture of the LLG allowed the lasers to be coupled easily. Aluminium mirrors (M1-M4) were used to steer the laser beam. M3 was mounted on a translation stage to allow changing the UVC radiation wavelength incident on the samples.

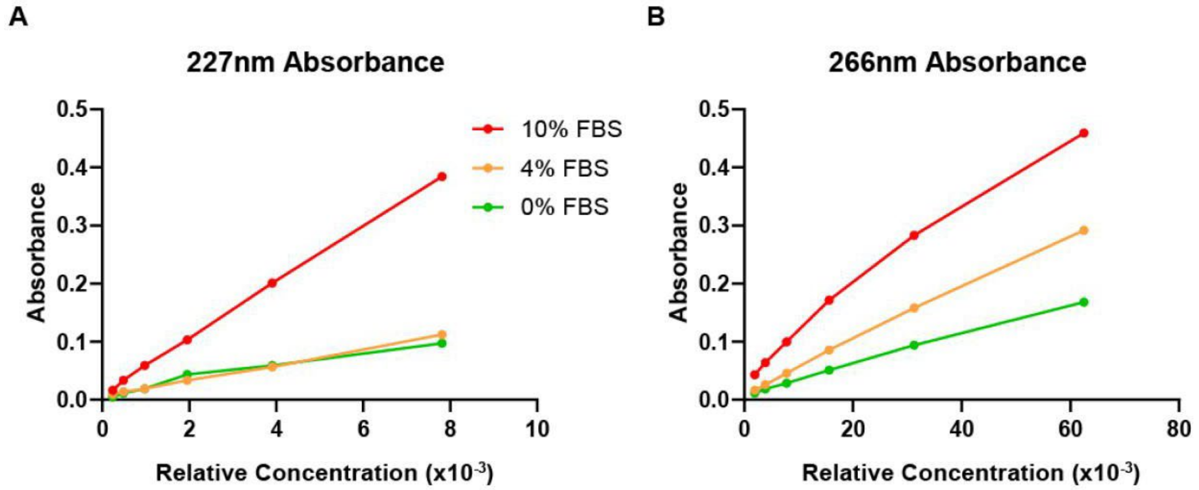


**Supplementary Figure 14. Absorbance of DMEM at 227nm and 266nm.** Absorbance at 227nm and 266nm of DMEM supplemented with 0%, 4% and 10% Foetal Bovine Serum (FBS). Molar extinction coefficients (ε) calculated from this were used to calculate the effective dose received through a sample of a given thickness.

## References

(1) Horcas, I.; Fernández, R.; Gómez-Rodríguez, J. M.; Colchero, J.; Gómez-Herrero, J.; Baro, A. M. WSXM:: A software for scanning probe microscopy and a tool for nanotechnology. *Rev Sci Instrum* **2007**, *78* (1). DOI: Artn 013705 10.1063/1.2432410.
(2) Zhang, D. M.; Mrozek, M. F.; Xie, Y.; Ben-Amotz, D. Chemical segregation and reduction of Raman background interference using drop coating deposition. *Appl Spectrosc* **2004**, *58* (8), 929-933. DOI: Doi 10.1366/0003702041655430.
(3) Ortiz, C.; Zhang, D. M.; Xie, Y.; Ribbe, A. E.; Ben-Amotz, D. Validation of the drop coating deposition Raman method for protein analysis. *Anal Biochem* **2006**, *353* (2), 157-166. DOI: 10.1016/j.ab.2006.03.025.
(4) Devitt, G.; Rice, W.; Crisford, A.; Nandhakumar, I.; Mudher, A.; Mahajan, S. Conformational Evolution of Molecular Signatures during Amyloidogenic Protein Aggregation. *Acs Chem Neurosci* **2019**, *10* (11), 4593-4611. DOI: 10.1021/acschemneuro.9b00451.
(5) Trevisan, J.; Angelov, P. P.; Scott, A. D.; Carmichael, P. L.; Martin, F. L. IRootLab: a free and open-source MATLAB toolbox for vibrational biospectroscopy data analysis. *Bioinformatics* **2013**, *29* (8), 1095-1097. DOI: 10.1093/bioinformatics/btt084.
